# The Hoard of the Rings. “Odd” annular bread-like objects as a case study for cereal-product diversity at the Late Bronze Age hillfort site of Stillfried (Lower Austria)

**DOI:** 10.1371/journal.pone.0216907

**Published:** 2019-06-05

**Authors:** Andreas G. Heiss, Ferran Antolín, Marian Berihuete Azorín, Benedikt Biederer, Rudolf Erlach, Niki Gail, Monika Griebl, Robert Linke, Michaela Lochner, Elena Marinova, Daniel Oberndorfer, Hans-Peter Stika, Soultana Maria Valamoti

**Affiliations:** 1 Austrian Archaeological Institute (ÖAI), Austrian Academy of Sciences (ÖAW), Wien/Vienna, Austria; 2 Integrative Prehistory and Archaeological Science (IPAS/IPNA), University of Basel, Basel, Switzerland; 3 Institute for Botany (210), University of Hohenheim, Stuttgart, Germany; 4 Institute for Oriental and European Archaeology (OREA), Austrian Academy of Sciences (ÖAW), Wien/Vienna, Austria; 5 Institute of Art and Technology, University of Applied Arts Vienna, Wien/Vienna, Austria; 6 Referat Naturwissenschaftliches Labor, Federal Monuments Authority Austria (BDA), Wien/Vienna, Austria; 7 Laboratory for Archaeobotany, State Office for Cultural Heritage Baden-Württemberg, Gaienhofen-Hemmenhofen, Germany; 8 Lira Laboratory, Department of Archaeology, School of History and Archaeology, Aristotle University of Thessaloniki, Thessaloniki, Greece; 9 Center for Interdisciplinary Research and Innovation (CIRI-AUTH), Aristotle University of Thessaloniki, Thessaloniki, Greece; Murdoch University, AUSTRALIA

## Abstract

Cereals, in addition to being a major ingredient in daily meals, also play a role in the preparation of foodstuffs for ritual purposes. This paper deals with finds that may correspond to such ritual preparations retrieved from the hillfort site of Stillfried an der March. The site, spreading across an area of ca. 23 ha, held a very important position among settlements of Late Urnfield period (particularly during the 10th– 9^th^ c. BCE), acting as a central place where large scale storage of grain as well as textile and metal production took place under the control of local elites. Three incomplete ring-shaped charred organic objects, found together with 14 rings and ring fragments made of clay were discovered in a secondary filled silo pit, excavated among a total of about 100 pits of this kind at the site. The overall good state of preservation of the organic ring fragments suggests that they were deposited intact on the bottom of the pit and covered well so that no re-deposition or damage occurred. This could be indicate their intentional placement in this position. Light and scanning electron microscopy revealed that the charred organic rings are cereal products containing hulled barley and a wheat species. Indications that the objects were shaped from a wet cereal mixture and had been subsequently dried without baking are discussed, as well as the possible significance of the find assemblage. The annular objects are put in context with the contemporary cereal spectrum as well as other cereal preparations from Stillfried, outlining their different *chaînes opératoires* for handling cereal food.

## Introduction

The importance of food, or rather, of different meals, for the constitution of individual and collective identities, and likewise as a means of social stratification, has long been a topic in the philosophy of culture and in social and historical anthropology [1–3], becoming a key topic in archaeology with ever-increasing importance [4–6]. However, in stark contrast to the vast evidence on crop diversity [7, 8], the scarcity of archaeological finds of food preparations and the even scarcer evidence on their ways of production has left a large component of past meals poorly understood and investigated, while conclusions from the archaeobotanical side had to remain on the level of anecdotic evidence. Luckily, interdisciplinary research into food transformation has gained momentum recently [9–11] and, in particular for south-eastern Europe, the awareness of the significance of research into the diversity of cereal-based dishes in the prehistoric archaeological record has been substantially improved [12, 13].

The cultural importance of the transformation of raw ingredients into food has probably been emphasised best by Sherratt, stating that “people do not eat species, they eat meals” [4]. The choices of plant species, their respective parts to be consumed [14–16] and the transformation of those into meals involves their incorporation into the social space of a civilization. Each of those meals reflects individual life experiences, collective memory, and identity [5, 17–19]. By human action and creativity, ecofacts [20] are transformed into artefacts [21–23] of defined structures and shapes in specific *chaînes opératoires* [24, 25]. Archaeological remains of meals in whatever form shall therefore clearly be regarded as parts of a material culture [26], requiring no less concise classification criteria and typologies than artefacts made of metal, ceramics, or glass [27, 28].

The archaeological remains of supposed “everyday food” and the insight into their composition and ways of production can tell us a lot about the nutrition value and sensory quality of this food, as well as the kind of tools involved and the amount of time invested in its production [10, 29, 30]. Altogether, these sets of information may also allow for conclusions about the social significance of the food preparations concerned. Yet when interpreting food remains from certain–or at least presumable–cultic contexts, understanding their sociocultural role is only possible when understanding respective *chaînes opératoires* involved in their production. Ethnography and history demonstrate how even a single aspect in either the components or processing can be decisive for the role of a food preparation in a particular rite: again taking fermentation as an example, leavening would be unthinkable in the *matzah* bread of Jewish Passover rites [31, 32], just as it would be for the altar bread of the Catholic Eucharist [33]. Concerning components, the Roman *mola salsa* [34] should certainly not be made of barley, while most Ancient Greek votive breads or cakes such as the *megalomazos* and the *hygieiai* should certainly be barley-based [35, 36]. Until now, only a few in-depth archaeobotanical studies have been able to contribute to such aspects of the respective ritual, the gestures and the knowledge behind it, and their embodiment in the final offerings [26, 30, 37].

### Research goals

Systematised scientific analyses of charred annular organic finds from a Late Bronze Age context were carried out in order to elucidate their composition and the processes involved in their production in order to test the hypothesis of them representing food in the widest sense. The results shall then be set in relation to extant data on remains of food plants and foodstuffs. The possible roles and significances of the three annular objects shall be elaborated against the background of the find context and its depositional history. Finally, the finds will be put within the wider context of the central European Late Bronze Age in regards to food processing practices and the role of food preparations in ritual activities. Purposes of food preparations other than consumption shall be discussed briefly.

### The site

**Fig 1:**
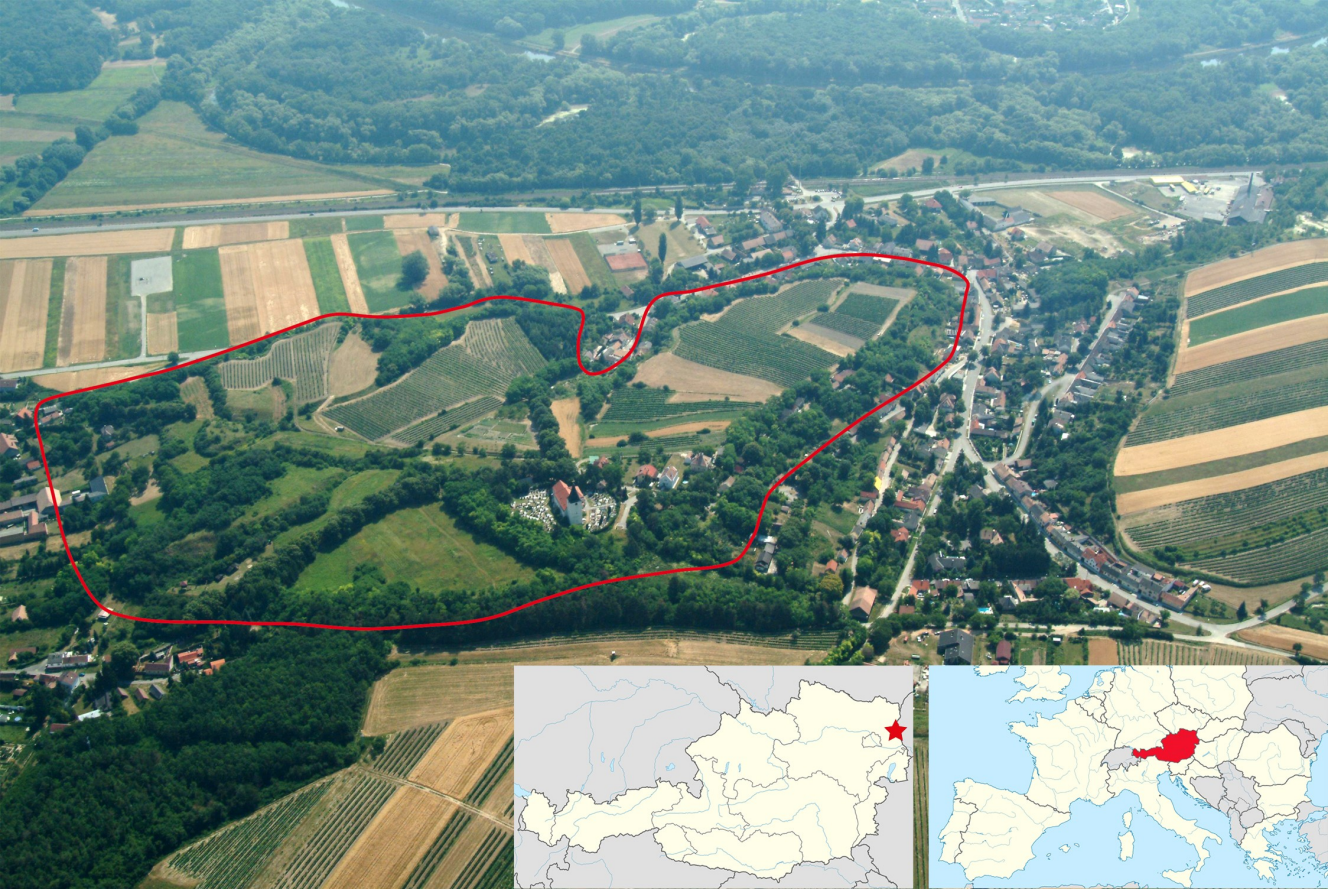
Aerial view of Stillfried an der March, as seen from the west. River March/Morava is visible in the upper part of the image. The reconstructed former fortification ramparts of the late Bronze Age hillfort are outlined in red. Insets indicate the location of the site at the Austrian-Slovakian border, and Austria’s location within Europe. Aerial Image No. 02030701.096 reprinted under a CC-BY license with permission from the Aerial Archive, Department of Prehistoric and Historical Archaeology, University of Vienna. Map sources: Wikimedia Commons, users TUBS and NordNordWest, CC BY-SA 3.0.

The hillfort site of Stillfried an der March is situated on a prominent knoll oriented in a north-south direction on the border between the gentle hills of the Weinviertel to the west, and the lowlands to the east, overlooking the banks of river March/Morava (Fig 1). The settlement shared its fortification and its strategic elevated position with other settlements of the Middle Danubian Urnfield culture [38, 39], yet was outstanding due to its location at the crossroads between the later so-called “amber road” running north-south, and a trade route running east-west leading towards the Little Carpathians.

**Fig 2:**
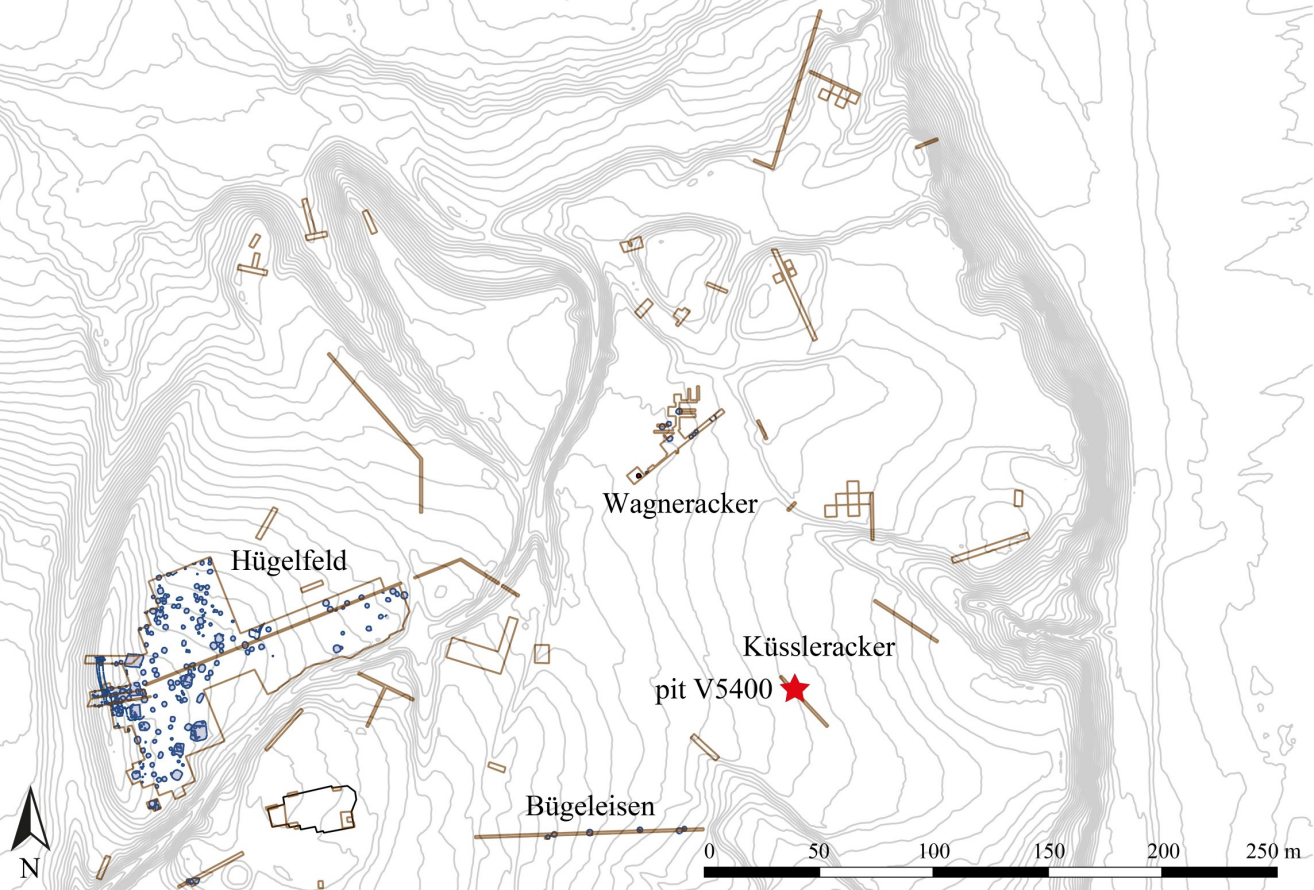
Elevation map of the northern area of the Late Bronze Age hillfort, indicating the location of pit V5400. Brown rectangles mark excavation trenches while blue lines indicate excavated structures. Toponyms indicated (e. g. Küssleracker, Wagneracker) are referred to in the text. Image: ÖAW-OREA / B. Biederer, I. Hellerschmid, I. Petschko.

About 2.5% of the 22 ha (220,000 m^2^) plateau has been excavated by the University of Vienna continuously from 1969 until 1989 [38]. From the Late Bronze Age, a large number of pits (about 100) were found distributed relatively evenly across the excavated parts of the hillfort (Fig 2). These share a general common shape of truncated cones, but vary in volume from 2 to 10 m^3^. They are interpreted as former storage pits for grain, and it is assumed that most of them were used repeatedly. None of the excavated storage pits contained major amounts of refuse as would have been the case had they been used for waste disposal [40], they had obviously been filled with rather homogeneous soil within short periods. Still, their excavation yielded a relatively large number and diversity of objects, often in unusual states or combinations: in at least two of the pits, human skulls were deposited [41] as were seven dead bodies in another pit [42, 43]. Eighteen terminated storage pits contained the deliberately deposited carcasses of domestic animals, as well as apparently tamed wild animals, some of which had died of old age such as wolves, hares and red deer [44]. Finds of daub fragments in some of the pits indicate dwelling houses nearby, but the connection between houses and pits is unknown: only very few postholes were found as the terrain has undergone severe ablation, most probably during the Middle Ages, only preserving the deeply dug pits.

Following D. Garrow’s definitions, most of these find assemblages correspond to “odd deposits” [45], which are outlined by one or more of the following characteristics: a) an extraordinary selection of finds, b) an exceptional state of preservation, c) a remarkable number or combination of finds, or d) exceptional find situations within a deposit or an unusual location of the find assemblage itself. These “odd” deposits from the secondary fillings of the storage pits probably cannot be understood without implying ritualised processes for placing those objects in the pits [42, 46–48].

### Food plants and plant foods from the settlement

**Fig 3:**
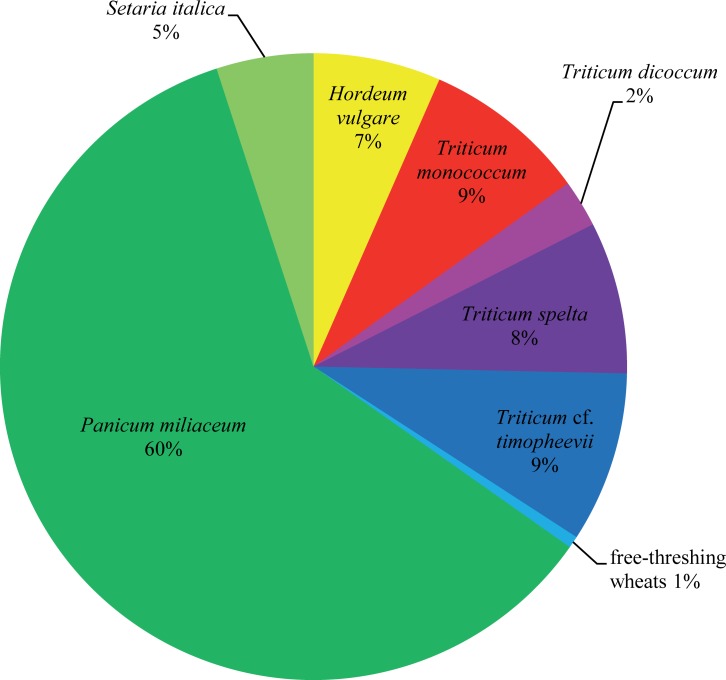
Simplified spectrum of cereal grain finds at Late Bronze Age Stillfried. The original data of M. Kohler-Schneider [49] were processed following the scheme of Stika & Heiss [52, 53]: included are only grain finds of taxa definitely or at least probably (cf.) identified to species level. Chaff finds as well as grains identified only to genus level are excluded, as are intermediate groups. Naked wheats which are not satisfactorily discernible by their grains are treated as a single species. Percentages are rounded to whole numbers. N = 4,256. Image: ÖAW-ÖAI / A. G. Heiss.

Extensive archaeobotanical analyses on samples from various archaeological contexts in the hillfort site, mostly the above-mentioned storage pits, but also from ovens and vessel contents, have resulted in a wide spectrum of crop plants [49–51]. As also observed in other sites of the region during Late Bronze Age [52, 53], the cereal spectrum is strongly dominated by broomcorn millet (*Panicum miliaceum*) comprising ca. 60% of all identified cereal grains (Fig 3), followed by more or less equal quantities (7–9% each) of hulled barley (*Hordeum vulgare*), einkorn (*Triticum monococcum*), spelt (*T*. *spelta*), and the “new” glume wheat most probably corresponding to modern Sanduri wheat (*T*. cf. *timopheevii*) [54–56]. Foxtail millet (*Setaria italica*), emmer (*Triticum dicoccum*) and free-threshing wheats (*T*. *aestivum*/*T*. *durum*/*T*. *turgidum*) only occur in minor quantities.

Among the other domesticates found at Stillfried, only the remarkably early finds of domesticated grapevine (*Vitis vinifera* subsp. *vinifera*) shall be highlighted here, which are interpreted as imports from the Mediterranean [49].

Aside from seeds and fruits, these archaeobotanical analyses have also resulted in rich evidence of various charred cereal preparations (Table 1): fragments of porridge are documented, as are chunks of bread showing clear differentiation between crumb and crust. The third type of material (nicknamed “Hirsotto”) was identified as a porridge-like preparation of coarsely crushed grains of barley, broomcorn millet and rye brome (*Bromus secalinus*), and it has already been the research focus of experimental approaches in order to infer its possible production steps and taste [49, 57, 58].

**Table 1:** Known occurrences of cereal preparations in Late Bronze Age pits from Stillfried. Identifications by M. Kohler-Schneider [49]. The localities “Hügelfeld” and “Wagneracker” are indicated in Fig 2 and the radiocarbon dates obtained there are given in Table 2.

	Hügelfeld			Wagneracker			
	V887	V888	V949	V5001	V5003	V5005	V5006
porridge	+	+	+	+	+	+	+
bread	+	+	+	+	+	+	+
“Hirsotto”	-	-	-	+	-	-	+

### The find context

The finds analysed in the current paper originate from the secondary filling of a Late Bronze Age former grain storage pit (V5400) from the eastern part of the hillfort (Fig 2). It was already excavated in 1978, but will be published the first time within the course of the ongoing Austrian Science Fund (FWF) project “Resource management, power and cult in Stillfried?” (Project No. P 28005-G25).

**Fig 4:**
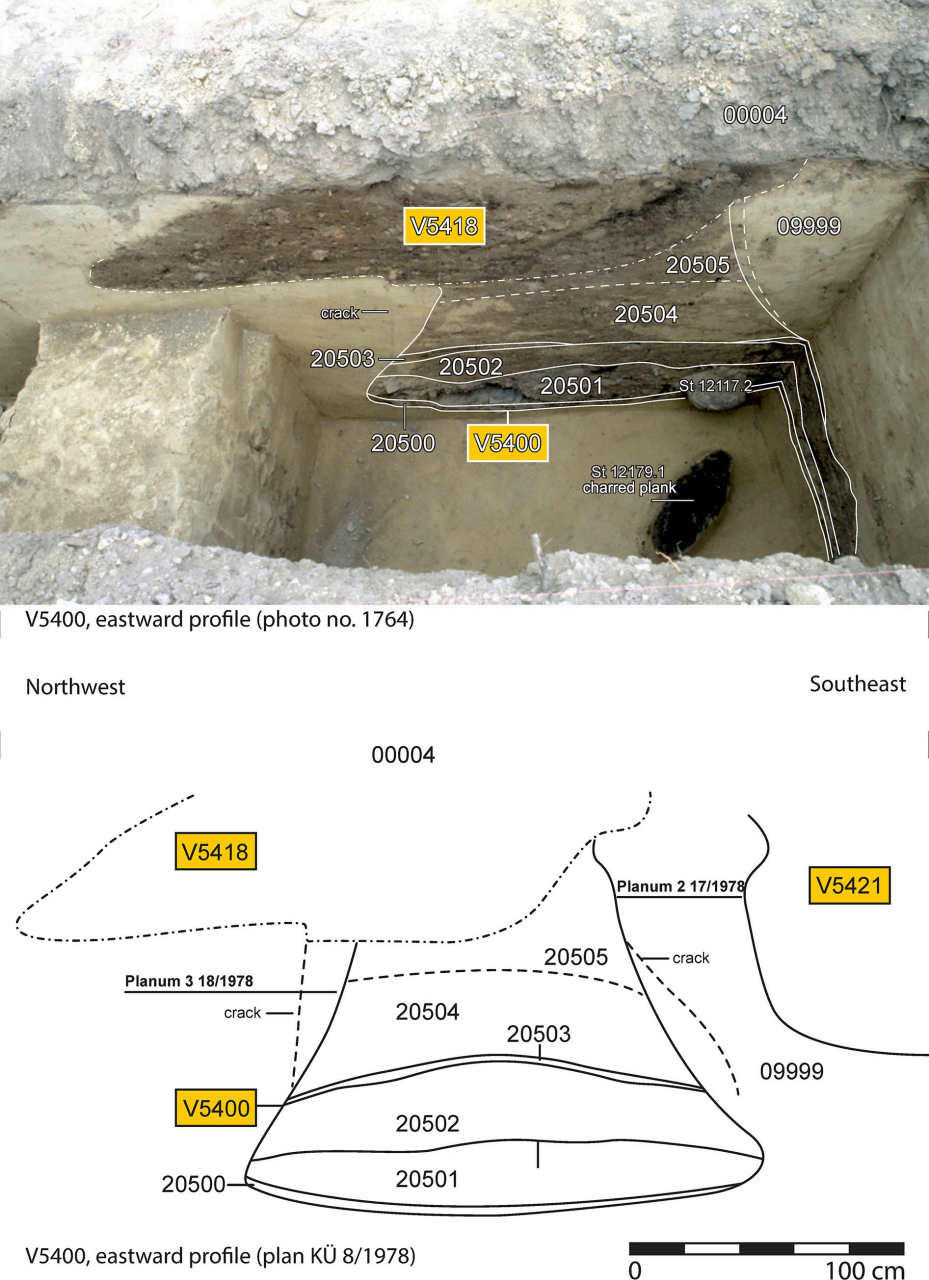
Annotated profile photo (above) and plan (below) through Pit V5400 at Küssleracker, 1978 excavation campaign. The grey layer at the bottom is the burnt debris which contained the rings. In the foreground, the charred plank (find number ST 12050.1) is visible. Image: excavation documentation Stillfried, State Collections of Lower Austria, Department of Prehistory, Protohistory, and Medieval Archaeology.

The structure is a 1.55 m deep pit, widening to a diameter of 2 m towards its bottom. Its volume of ca. 2.8 m^3^ lies in the lower range of the pits at Stillfried. In the basal layer, a charred wooden plank was found. The filling above (Fig 4) contained elements of a burnt-down house, such as a massive layer of burnt rubble with large pieces of daub (about 20 kg), charred wood and broken pottery. The latter is dominated by rough houseware (cooking pots, storage vessels, in total about 8 kg), most of the pieces bearing traces of fire. In contrast, the fine tableware from the same layer shows mostly no evidence of burning. A similar composition of the secondary filling as well as a similar state of preservation of the ceramics was found in storage pit V5001 of the same hillfort [47]. Typological dating of the ceramics is in congruence with Hallstatt B2 and B3, corresponding to c. 960–800 BCE [59].

**Fig 5:**
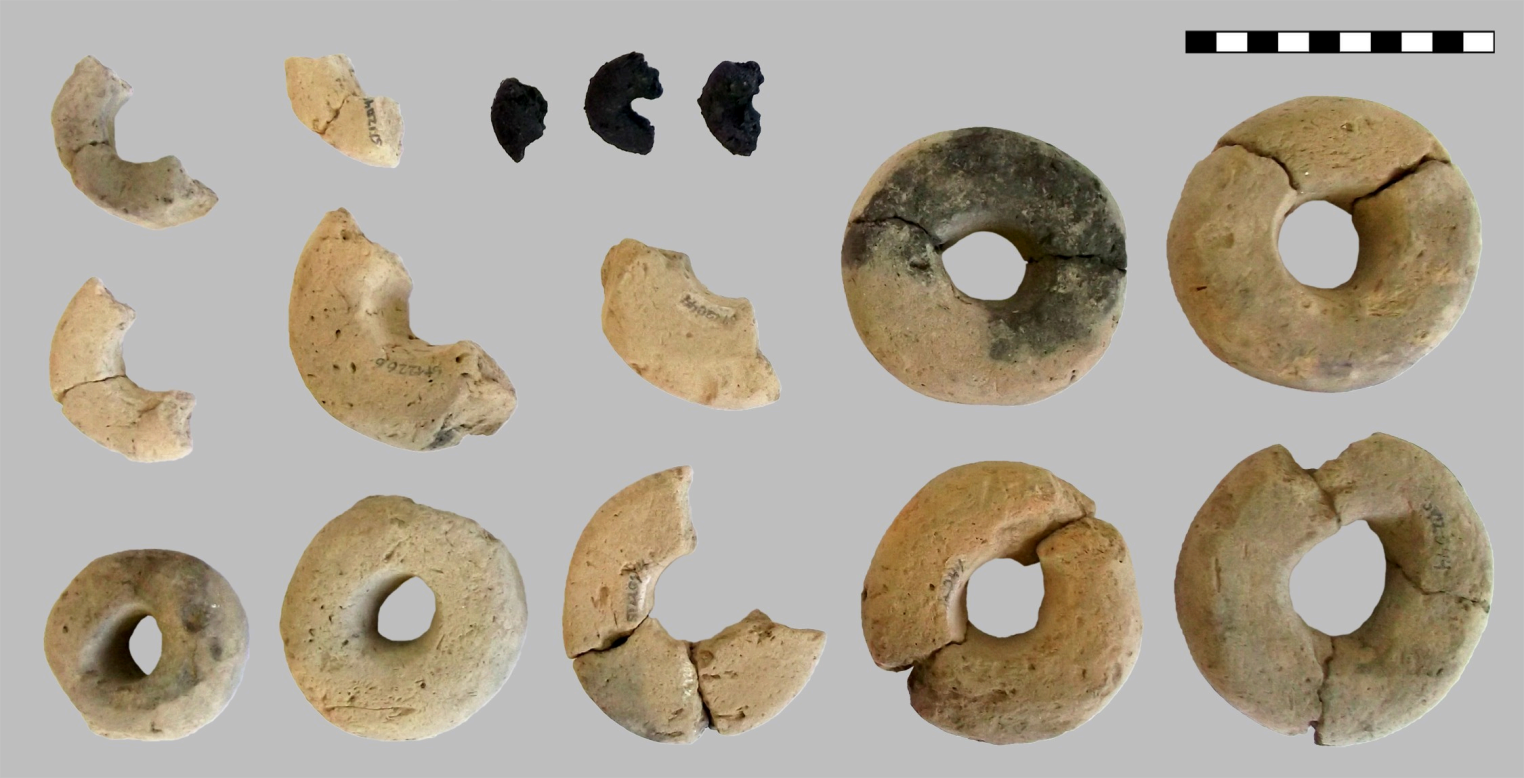
The annular objects from the find assemblage in the debris layer of pit V5400. Twelve of the 14 rings made of clay (find numbers ST 12044.1–9, 12116.1, 12266.1–2, box 10/60044) are shown, as well as the charred organic rings (find numbers ST 12050.2–4, box 9/60043) which are the focus of this contribution. Scale bar units: centimetres. Image: ÖAW-OREA / B. Biederer.

From the layer of burnt debris, 14 rings made of clay were recovered, as well as three fragments of rings made of charred organic matter, which are interpreted as originating from three individual rings (Fig 5). The clay rings have outer diameters of 8.6–10.5 cm and 6.2–7.7 cm. Two were found in complete state, another four can be reassembled completely from two or three pieces, while the remaining ones are fragmentary. These had been only weakly fired and had probably been used as loom weights [47], as is also indicated by traces of use. Similar clay rings are documented from pits V5003 and V5004.

**Fig 6:**
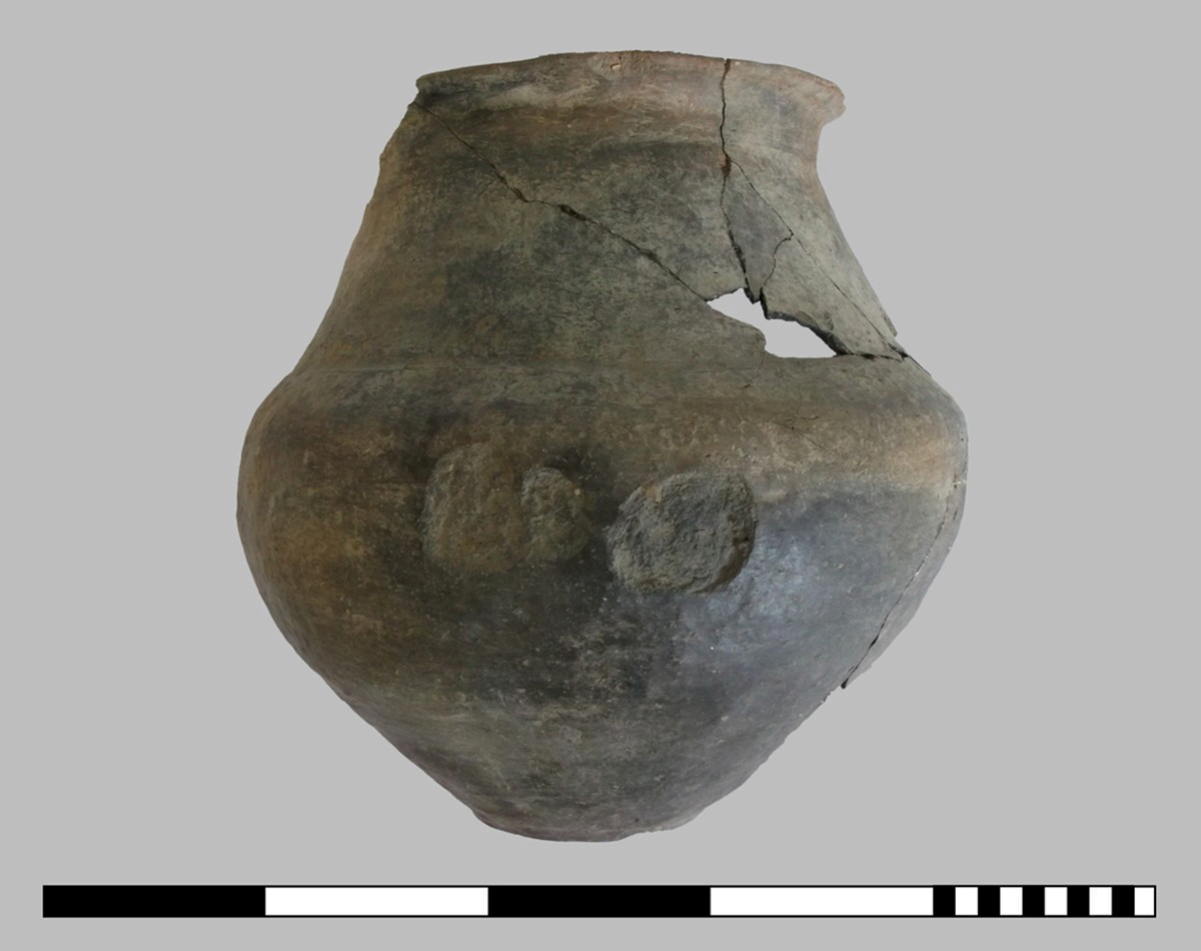
Vessel with conical neck (*Kegelhalsgefäß*) and handle from the burnt debris layer. This deviant ceramic object, split in half vertically (find number ST 12051.1/box 1/60001), was possibly used for scooping liquid. Scale bar units: decimetres/centimetres. Image: ÖAW-OREA / M. Griebl.

**Fig 7:**
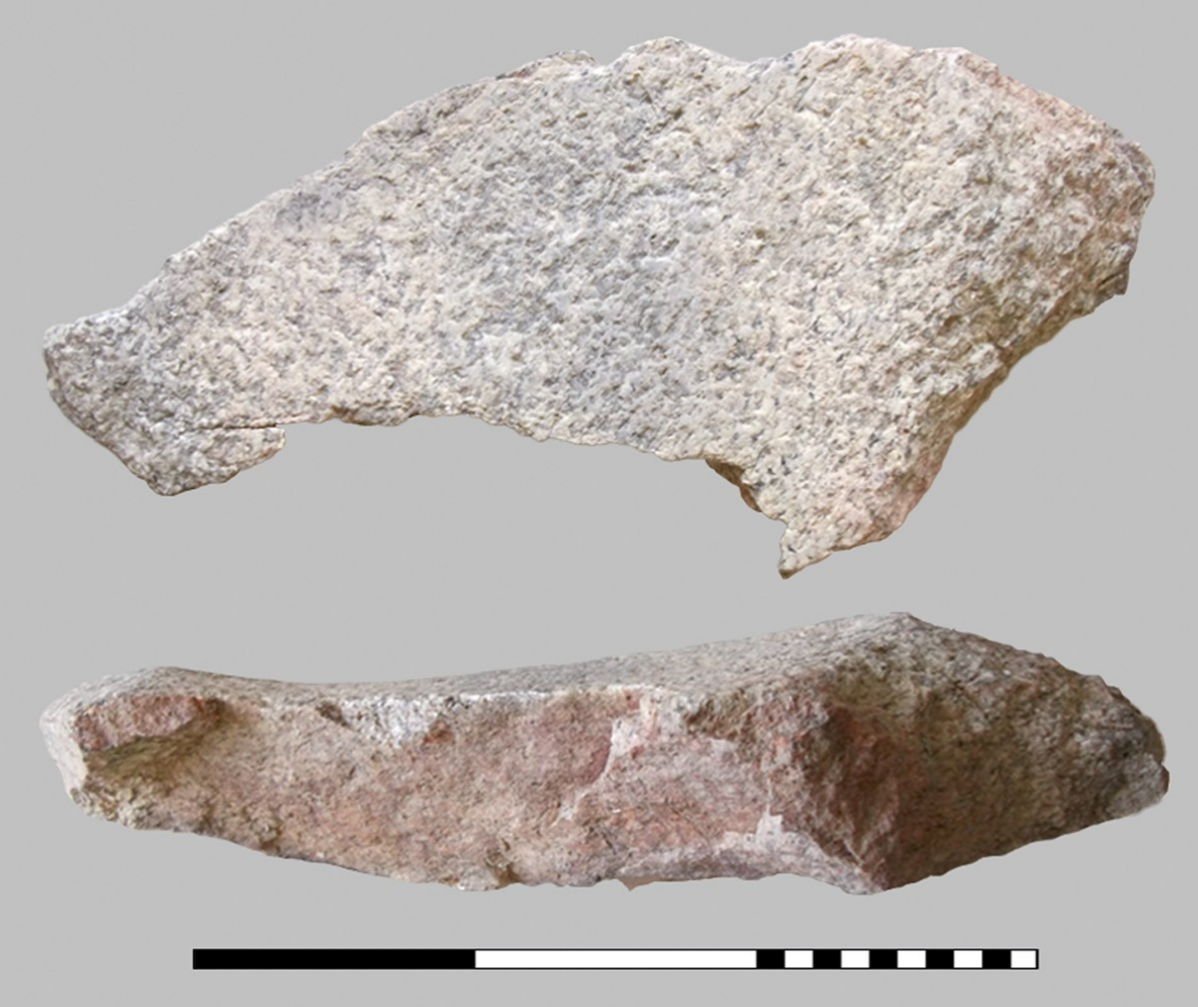
Edge piece of a grinding stone with preserved top surfaces and side edges, broken in halves. The marked burning traces of this object (find number ST 12260.5, box 3/60048) may derive from the same fire as the remaining burnt debris. Scale bar units: decimetres/centimetres. Image: ÖAW-OREA / B. Biederer.

**Fig 8:**
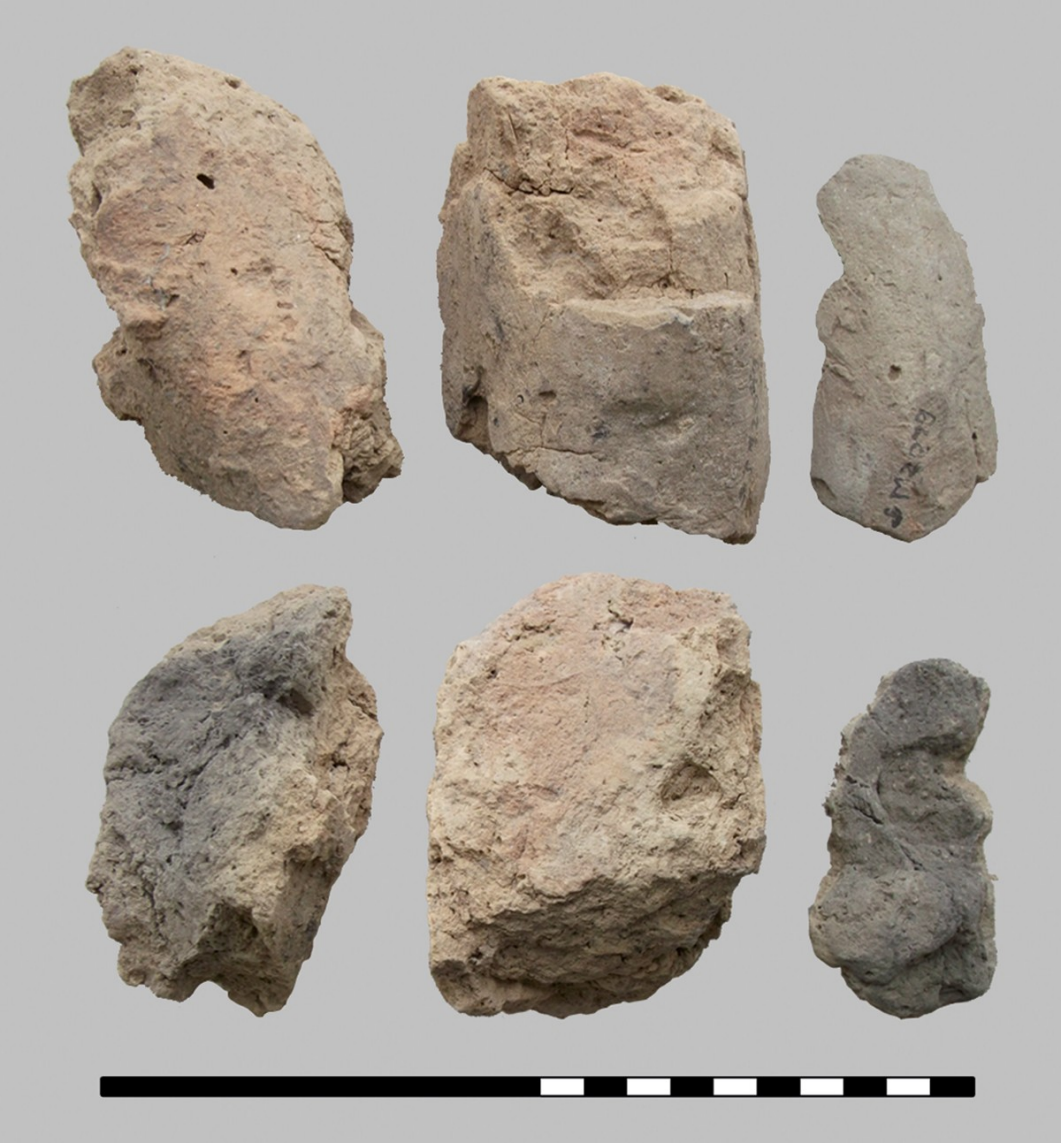
Burnt daub, interpreted as architectural elements. These elongated daub fragments (find number ST 12049.11, boxes 2/60028 and 2/60033) are comparable to finds out of the burnt rubble-layer in storage pit V5001 of the same hillfort. Scale bar units: decimetres/centimetres. Image: ÖAW-OREA / B. Biederer.

The three charred organic rings are significantly smaller, with outer diameters ranging from 2.6 to 3.6 cm. From the same layer, other interesting finds also came to the light: two deviant ceramic finds–a vertically halved vessel with a conical neck used for scooping liquid (Fig 6) and a stamp-like object made of a lid–as well as grinding stones (Fig 7), a bronze needle, a processed antler and daub fragments (Fig 8). Above this layer only a few finds were detected in a filling dominated by the local humus soils and loess [46, 47].

The overall composition of finds from pit V5400 clearly speaks against tertiary refuse, i.e. the diachronic accumulation of materials eventually constituting the pit’s filling [40], but rather indicates a singular event of deposition. Like in the other terminated storage pits mentioned above, the unusual combination of objects in a particular state qualifies the find assemblage as an “odd deposit” [45]. An intentional and deliberate (ritual) deposition of the pit’s contents shall therefore serve as a working hypothesis.

## Materials and methods

The three incomplete charred rings (Find Nos. ST 12050.2–4 / Box 6004) covered by the current study are the property of the Provincial Government of Lower Austria. Like the other mentioned finds from Stillfried, they are stored in the archaeological depot (Kulturfabrik), Donaulände 33, 2410 Hainburg an der Donau, Austria. The material is accessible on request for re-examination. Several of the mentioned archaeological finds are part of the permanent exhibition in the Museum Stillfried in Stillfried an der March [60]. No permits were required for the described study.

### Conservational considerations: Some challenges in the analysis of museal objects

The surfaces of all three charred organic rings exhibited an opaque and glossy appearance, apparently deriving from a consolidating agent applied immediately after excavation in the late 1970s. As the diagnostic features of the surface where not readable due to this treatment, the consolidant had to be removed to some extent in order to enable microscopical analysis. Preliminary tests showed a slight swelling in water while the application of a range of other polar and non-polar solvents did not show any effect. In order to gain detailed information on the composition of this consolidating agent, chemical analysis was carried out by means of FTIR (Fourier-Transform Infrared Spectroscopy). A portion of the swollen glossy coating of Sample A was taken, air-dried, and directly analysed in a diamond cell (transmission mode) using a Bruker Tensor 27, coupled with a Hyperion 2000 microscope, resolution: 4 cm^-1^, measurement range: 4000–550cm^-1^.

**Fig 9:**
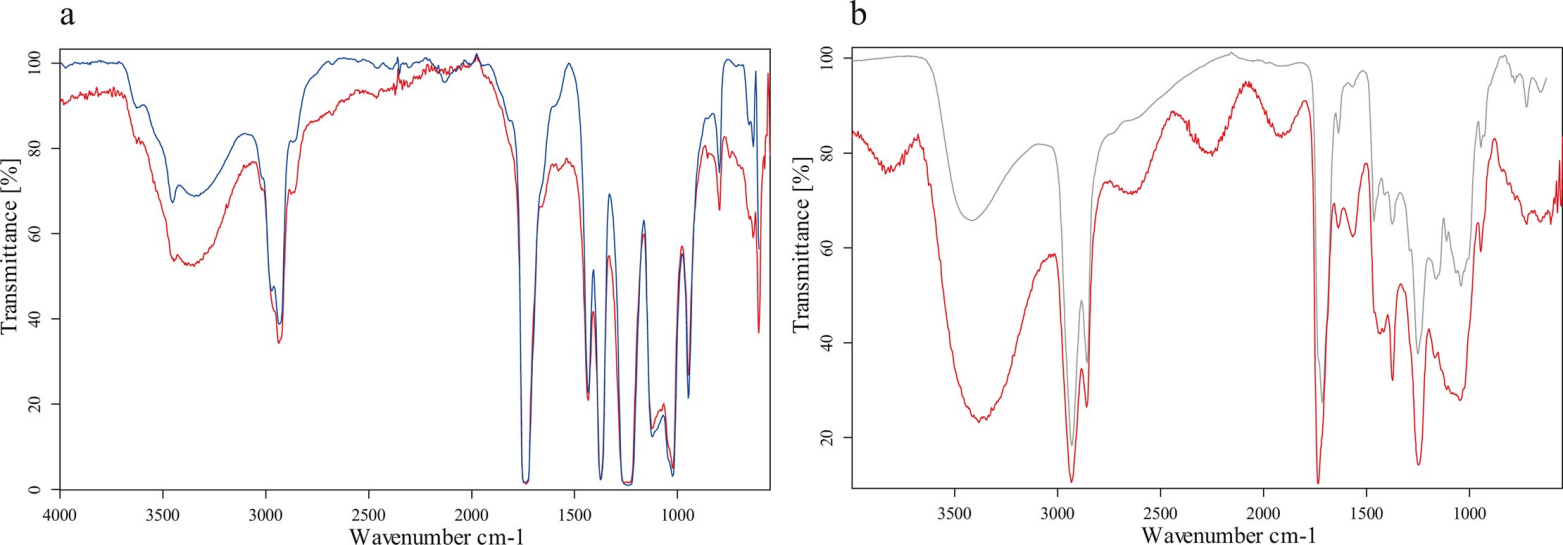
FTIR spectra of the surface coating of sample A. a) PVAC. red: sample curve, blue: reference material, b) Shellac. red: sample curve, grey: reference material. Image: BDA / R. Linke.

The results (Fig 9) show the presence of polyvinyl acetate (PVAC) [61], which is commonly used as wood glue and which has apparently been applied onto the find as consolidant. Contrary to a number of synthetic resins which were also in use during the 1970s, PVAC is soluble in aqueous solution as well as in some solvents and is therefore removable to some extent. Since its first reported use in conservation in 1932 [62], it has been in use as consolidant throughout the 20^th^ century for objects made of a broad range of materials including glass [63], ceramics [64], textiles [65], leather [66], paper [67], and wood [68]. It is still in use as a consolidant for archaeological finds [69–71], with a particular focus on the consolidation of human bone material [72–74].

In addition, traces of a translucent reddish brown coating were identified as shellac [61] which is interpreted as a secondary addition. Shellac has commonly been used as varnish for many centuries and is nowadays still used in conservation-restoration for special applications. Apparently shellac was still in use as consolidant in the 1980s in central Europe–despite the fact that some of its properties are highly problematic for this kind of application [75].

The observed low solubility of the consolidant on sample A may be linked to the ageing of the PVAC, as photochemical degradation leads to cross-linking and chain scission in the material, under release of volatile compounds [76]. Issues regarding the removal of PVAC are, however, not limited to conservation: Especially in the field of radiocarbon dating PVAC can be problematic [77, 78]. As the PVAC used for consolidation on Sample A was not sufficiently removable by solvent treatments but swellable with water, the decision was made to manually remove it from a part of the surface of Sample A after the application of water soaked cellulose fibres. The surface of the object showed enough strength to lift the softened consolidant with a set of very fine metal tools under the stereomicroscope. The fragments of the charred material (2.8 mg) which came off during this procedure were labelled sample B and kept for radiocarbon dating. As a consequence of the observed changes in surface caused by the removal of the consolidating agent, namely the loss of the glossy appearance, we decided to limit this procedure, and consequently in-depth analysis, to sample A.

Chemical analyses were not carried out in the current study due to the limited results to be expected in comparison to tissue-based approaches [9, 79, 80], as well as due to the known contamination of the finds with consolidating agents. In contrast to method sets involving chemical dissolution of the material [81–83], we followed a less invasive approach by identifying components via tissue remains visible on fractured faces as already established [10, 30]. Aside from the samples for SEM analysis and AMS dating, no fresh fractures were produced due to conservation considerations regarding the uniqueness of the finds.

### Radiocarbon dating

AMS dating was carried out at BETA Analytic, Miami. As a preventive measure against the above-mentioned organic contaminants, standard acid-alkali-acid treatment as well as solvent extraction were applied prior to dating. The results as well as all other radiocarbon dates mentioned in this article were calibrated with OxCal 4.3 [84], basing on the IntCal13 calibration curve [85].

### Microscopy and photography

Photo documentation was carried out with a Canon EOS 5DS R equipped with a Canon EF 100 mm/2.8 L Macro IS USM objective, using a Broncolor Siros 400 WiFi / RFS 2.1 illumination device in order to maintain constant colour temperature.

Light microscopic observations of the objects’ supposedly post-depositional fractured faces were carried out using integrated solutions by Olympus: an SZX-10 stereo microscope at magnifications up to 63-fold, and a BX53M metallurgical microscope with up to 500-fold magnification, both with integrated camera UC909 and the software Olympus Stream Basic, applying its Instant EFI (Extended Focal Imaging) image stacking function in order to improve depth-of-field of the photographed irregular surfaces.

The cleaned surfaces of ring A were additionally analysed using a Quanta 250 FEG environmental SEM in ‘Low Vacuum Mode’ and without prior sputter coating. We used acceleration voltages ranging from 5 to 15 kV in the generation of secondary electron images. Depth-of-field was enhanced in some cases by combining an image stack with the software Helicon Focus 7 [86].

### Experimental reference material

Some aspects of the analysis required experimentally charred reference material, in particular the preservability of starch granules in charred state and the formation of cavities during charring.

*Triticum spelta* ‘Bauländer Spelz’ was cultivated in autumn sowing by the Mechler family in Altheim-Walldürn, southwestern Germany, and harvested as *Grünkern*, i.e. in an unripe state [87] after nine months of growth. The grains were charred in a Nabertherm NA 15/65 muffle furnace under various charring regimes: at low temperature (230° C) for 7, 9, 12, and 24 hours [88], and at high temperature (300°C) for 6 hours.

Sprouted two-rowed hulled barley (*Hordeum vulgare* subsp. *vulgare* f. *distichon*) was acquired from the Durst Malz company, Bruchsal-Heidelsheim, Germany, in various states of germination (3 days and 5 days) and charred using the same muffle furnace under varying conditions (dry, soaked in water, and with water) at low temperature (230° C) for 24 hours [88].

SEM imagery for the *Grünkern* was produced using a Zeiss DSM 940 after sputter coating with gold/palladium in a Balzers SCD 040, for the barley malt with a JEOL JSM-6390LV after carbon coating using a JEOL-4X vacuum evaporator.

### Identification of plant components

The histological identification of cereal tissues is based on well-established plant anatomical criteria for the identification of bran and glumes. The criterial typically applied in food analysis [89–92] were adapted for archaeobotanical use [93–96]. We regarded the discriminatory power of cereal transverse cell wall thickness and pitting as limited to the genera *Triticum*, *Secale*, and *Hordeum*–with the concession of incomplete differentiation between barley and the millets–as laid out previously [10, 30].

### Measuring

Original diameters of the charred rings were reconstructed using the software ImageJ [97] by manually aligning ellipses to the outlines of each fragment. ImageJ was also used for the measurements of plant tissue fragments visible in the scanning electron micrographs of sample A, as well as in the light micrographs of all three rings. Pore/cavity size distributions were measured in light micrographs of all three rings following the methodology as laid out in [10], i.e. measuring pore sizes with ImageJ’s “fit ellipse” function on black and white photographs of the fractured faces (threshold: 64 out of 255 grayscale values). Obvious cracks were excluded from the measuring, as were Instant EFI-generated visual artefacts on the image margins. Likewise, pores smaller than 100 μm were not measured. The resulting values were evaluated in histograms with constant 100 μm class intervals. Fragments of cereal components were measured only for ring A, as this was the only sample where both light microscopy and SEM images were available. The results, presented in histograms (100 μm steps), are grouped according to modern equivalents of ground cereals [98, 99]: flour and dunst (particles smaller than 300 μm) vs. semolina (300–1000 μm) vs. grist (larger than 1000 μm).

### Terminology

As laid out previously [10], a precisely defined common terminology is still widely lacking in the analysis of archaeological “bread”-like objects. Building on current works on cereal-based foodstuffs, we suggest the very general definitions of “cereal preparation”, synonymous to “cereal product”, both following FAO suggestions [100], for any charred find bearing evidence of processed cereals. We suggest the term “bread-like object” for any archaeological find of a cereal product which shows traces of intentional shaping [10]. We suggest this term as a general working title preceding analysis, in order to avoid the use of expressions implicating certain *chaînes opératoires*.

In microstructure, we follow standard terminology in food technology by calling an airspace within a cereal preparation “pore” [101] or “cavity” [10]. The recently published term “close void” [102] may indeed refer to “closed pore” and “closed void” [103]–but is not appropriate in this context, as it refers to the interconnectivity of pores, which can neither be observed nor judged from the surface of such objects [104, 105]. A pore not exceeding 200 μm in diameter is named “micropore” following the suggestion by González et al. [102]. In contrast to previous work [30], we suggest a terminological delimitation between a “crack” as a taphonomical feature, either caused by charring itself or by mechanical stress after charring, and a “furrow” for the elongated hollow spaces with irregular margins which are features of the dough/batter itself and often intergrade with pores. Unless a definition of the term “channel void” [102] becomes available–it may be synonymous with either or both of the aforementioned–so far its use should be avoided.

## Results

### Dating

The date range proposed in the introduction (c. 950–800 BCE) could be further narrowed down by the presence of the older type of vessels with conical necks (*Kegelhalsgefäße*) with a handle [59], in combination with the AMS dating of sample B (Table 2). Altogether, we propose dating the context to Ha B2 (960–900 BCE).

**Table 2:** Radiocarbon dates of the contexts discussed in the text.

Pit number	Laboratory no.	^14^C Age	δ^13^C (‰)	Calibrated age ranges (2σ)
V5400 (ST 12050.4 B)	Beta-486379	2780 ± 30 BP	-25.0	1004–844 cal BCE (95.4%)
* V5001	ETH-16498	2880 ± 50 BP	-22.0 ± 1.1	1210–926 cal BC (95.4%)
* V5003	ETH-16497	2750 ± 50 BP	-25.3 ± 1.1	1005–811 cal BC (95.4%)
* V5005	ETH-16499	2830 ± 50 BP	-22.1 ± 1.1	1126–844 cal BC (95.4%)
* V5006	ETH-16500	2840 ± 50 BP	-22.9 ± 1.1	1131–893 cal BC (91.1%)

Samples preceded by an asterisk * come from a previous publication [49]. Pit numbers used in the current publication correspond to those published in 2001 as follows: V5001 = A3/1; V5003 = A/0 18; V5005 = Schnitt 7 Verfärbung 2; V5006 = Schnitt 7 Verfärbung 5. For this publication, all data were (re-)calibrated with OxCal 4 [84] using the IntCal13 calibration curve [85].

### General traits

**Fig 10:**
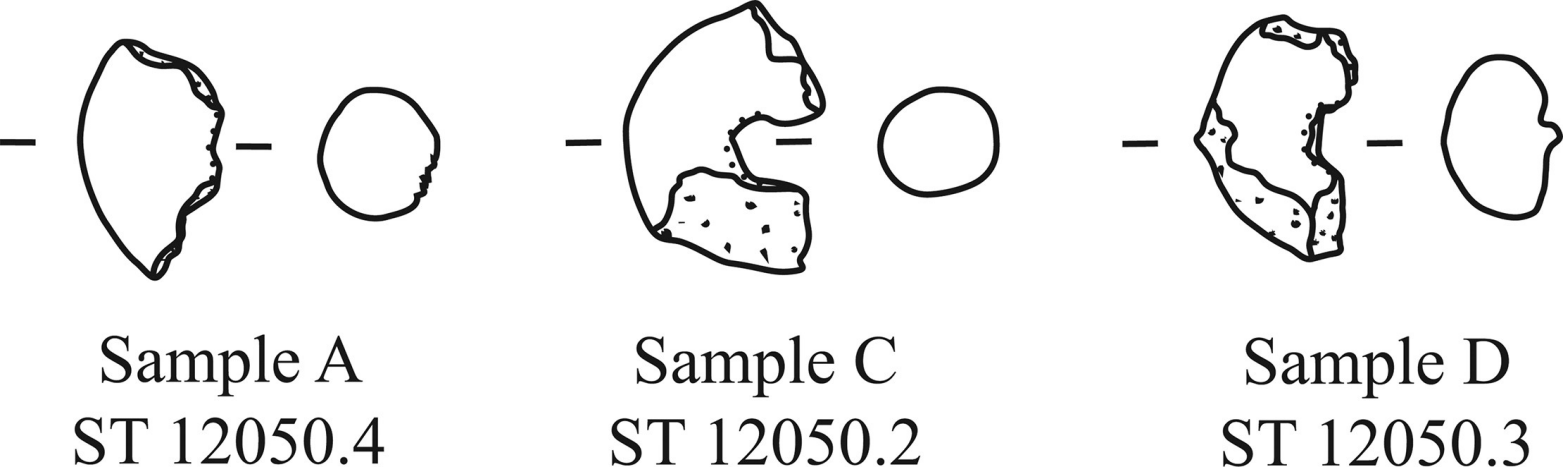
Line drawing of the three charred organic rings. Image: ÖAW-OREA / B. Biederer, S. Tikatsch.

**Fig 11:**
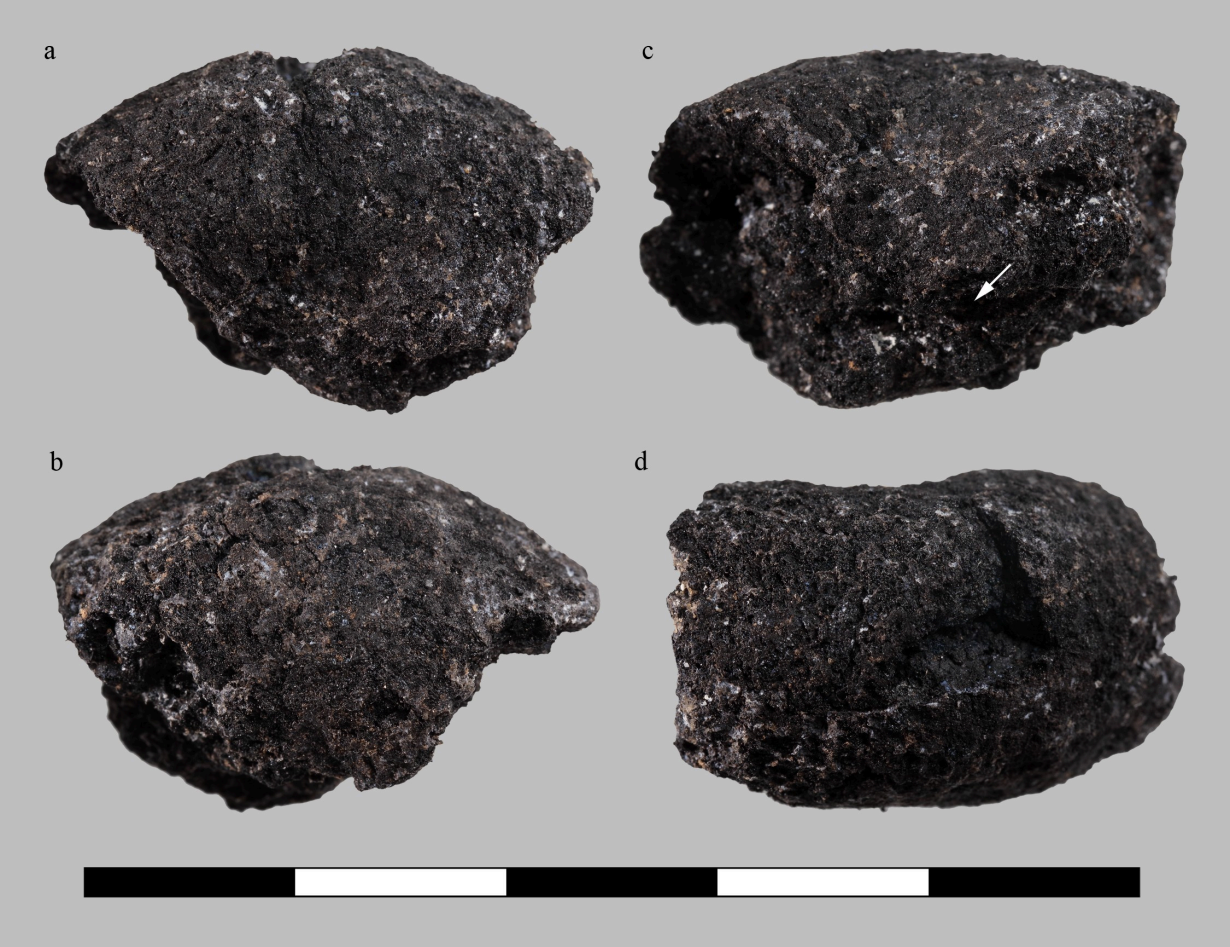
Charred organic rings, sample A (find no. ST 12050.4). **a** “top”, **b** “bottom”, **c** inside, **d** outside view. Arrows indicate furrows extending to the surface. Scale bar units: centimetres. Image: ÖAW-ÖAI / N. Gail.

**Fig 12:**
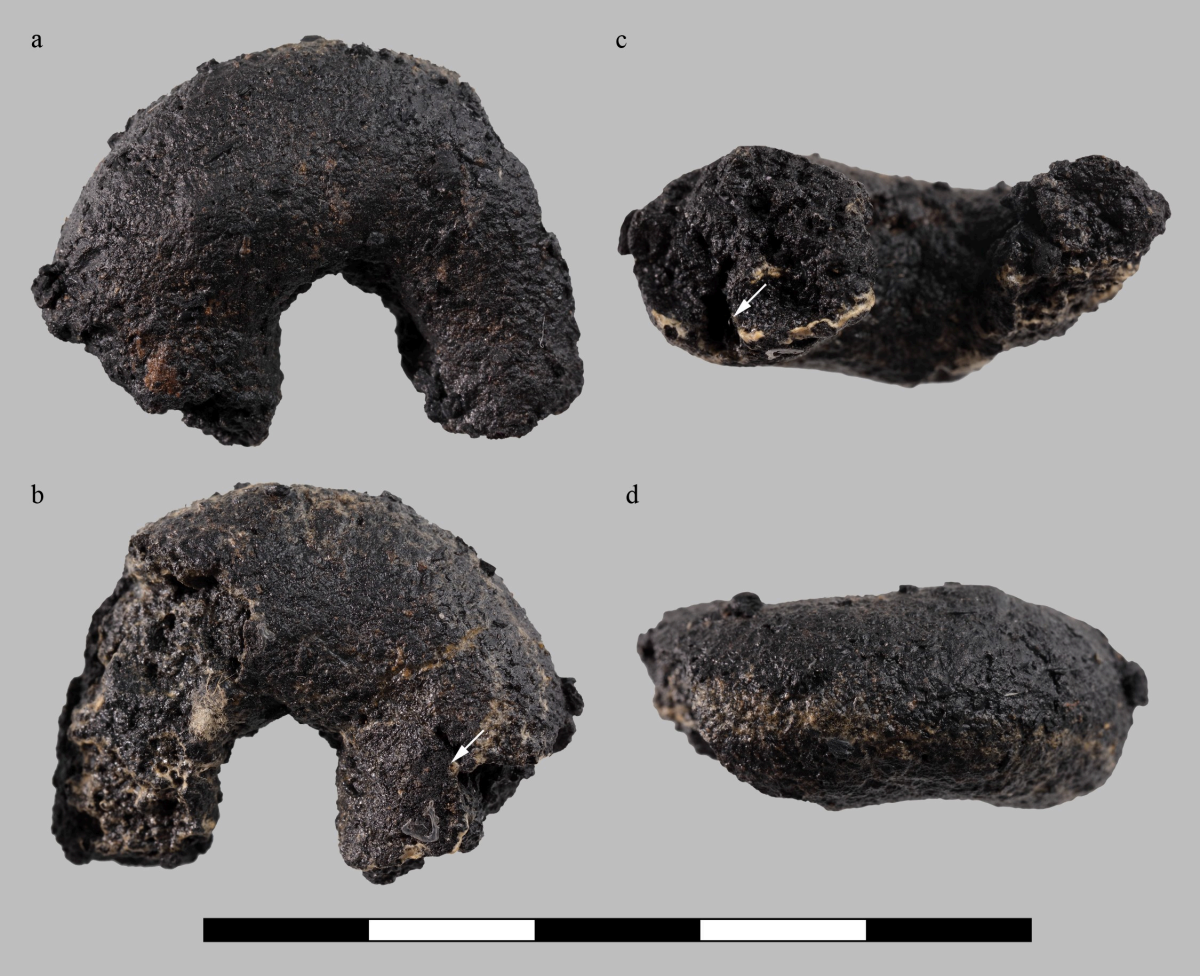
Charred organic rings, sample C (find no. ST 12050.2). **a** “top”, **b** “bottom”, **c** inside, **d** outside view. Bright surface features originate from the consolidating agent (wood glue). Arrows indicate furrows extending to the surface. Scale bar units: centimetres. Image: ÖAW-ÖAI / N. Gail.

**Fig 13:**
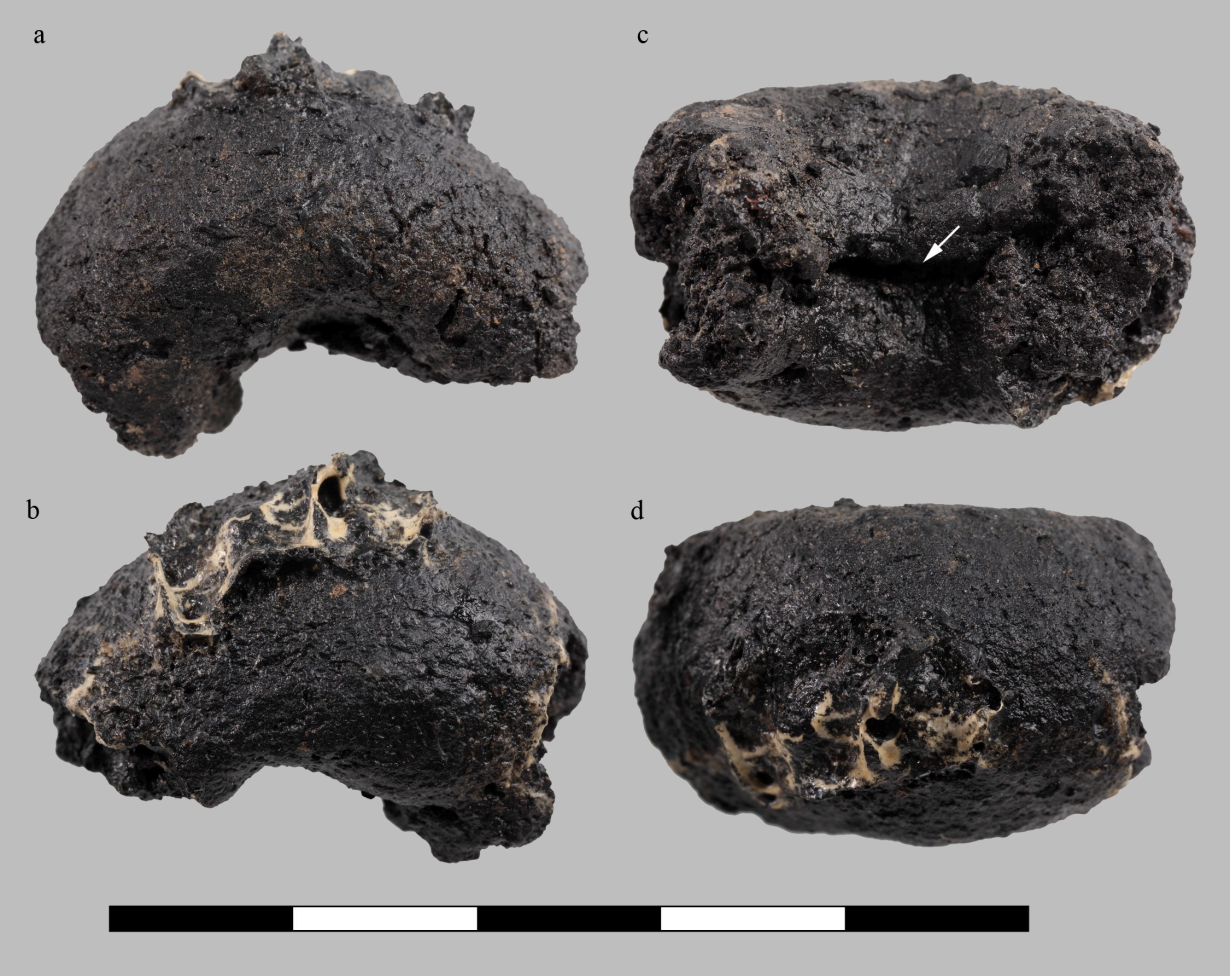
Charred organic rings, sample D (find no. ST 12050.3). **a** “top”, **b** “bottom”, **c** inside, with visible furrow, **d** outside view. Bright surface features originate from the consolidating agent (wood glue). Arrows indicate furrows extending to the surface. Scale bar units: centimetres. Image: ÖAW-ÖAI / N. Gail.

Each of the three samples is interpreted as a fragment of an originally annular, or rather torus-shaped object (Fig 10, Fig 11, Fig 12, Fig 13). All three rings are roughly of the same size, shape, and proportions, their original diameters are reconstructed as ranging from 26 to 36 millimetres (Table 3). Apart from the flaky protrusions caused by the consolidating agent still adhering to samples C and D, their surfaces are all smooth and inconspicuous. Neither particular decorations nor fingerprints could be observed, only sample D shows a thin overlaying structure (Fig 13A) on the outer side, as well as a marked furrow on the inner side (Fig 13C).

**Table 3:** General traits of the three annular objects.

	Sample A	Sample C	Sample D
	(Fig 11)	(Fig 12)	(Fig 13)
Shape	torus (^1^/_4_)	torus (^1^/_2_)	torus (^1^/_3_)
Ring thickness, range [mm]	12.1–15.8	10.1–14.2	11.2–18.9
Outer diameter, range [mm]	26–36	31–33	27–36
Ratio max. diameter : max. height/thickness	2.28 : 1	2.32 : 1	1.9 : 1
Weight [g]	1.37	2.45	3.01

**Fig 14:**
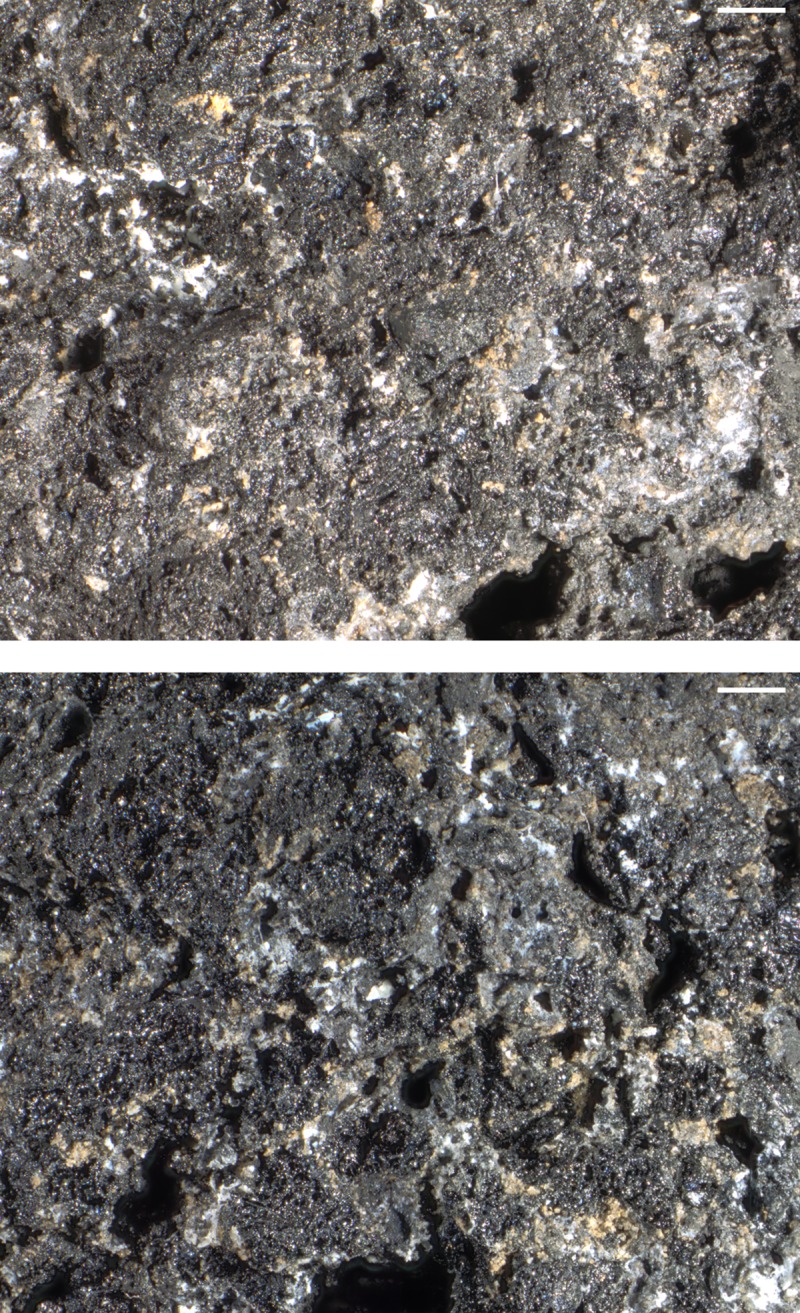
The two fractured faces of sample A under the binocular (10x). Scale bar lengths: 1 mm. Image: ÖAW-ÖAI / A. G. Heiss.

**Fig 15:**
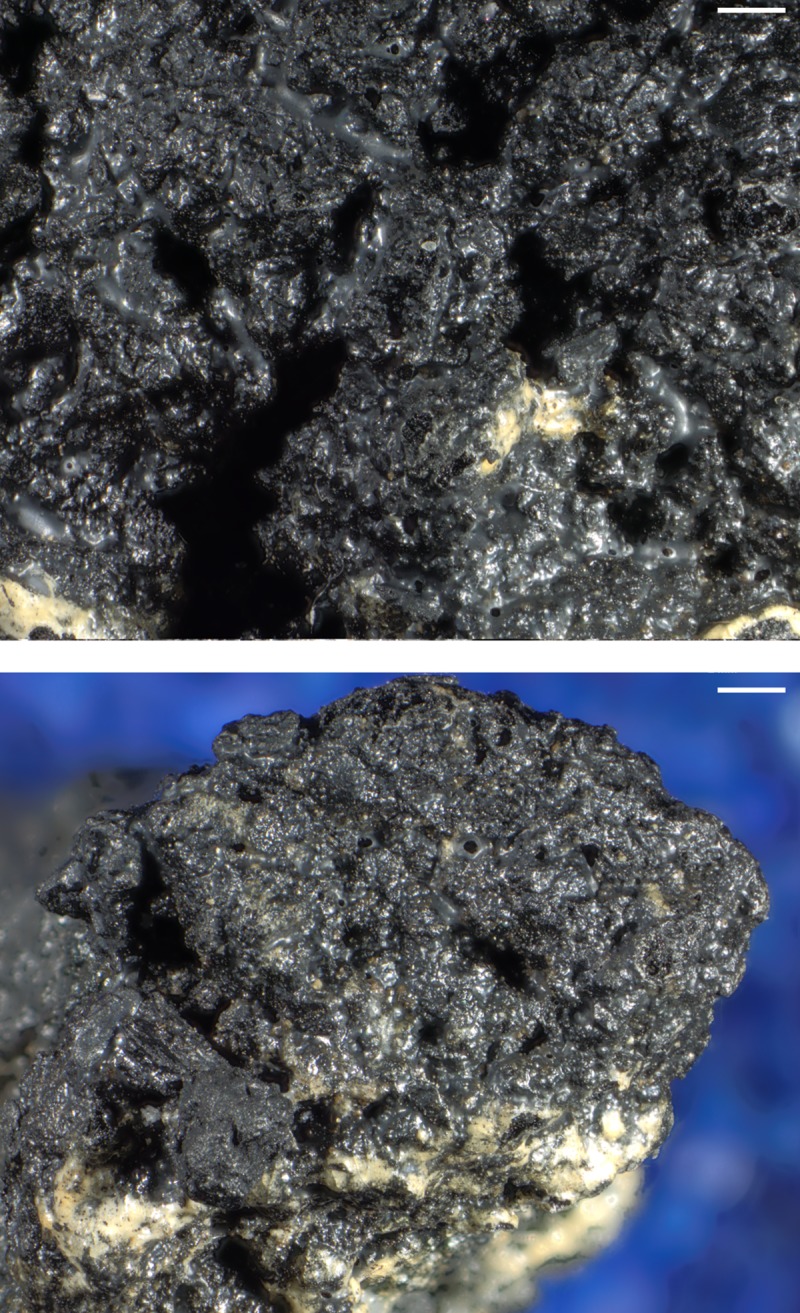
The two fractured faces of sample C under the binocular (10x). Scale bar lengths: 1 mm. Image: ÖAW-ÖAI / A. G. Heiss.

**Fig 16:**
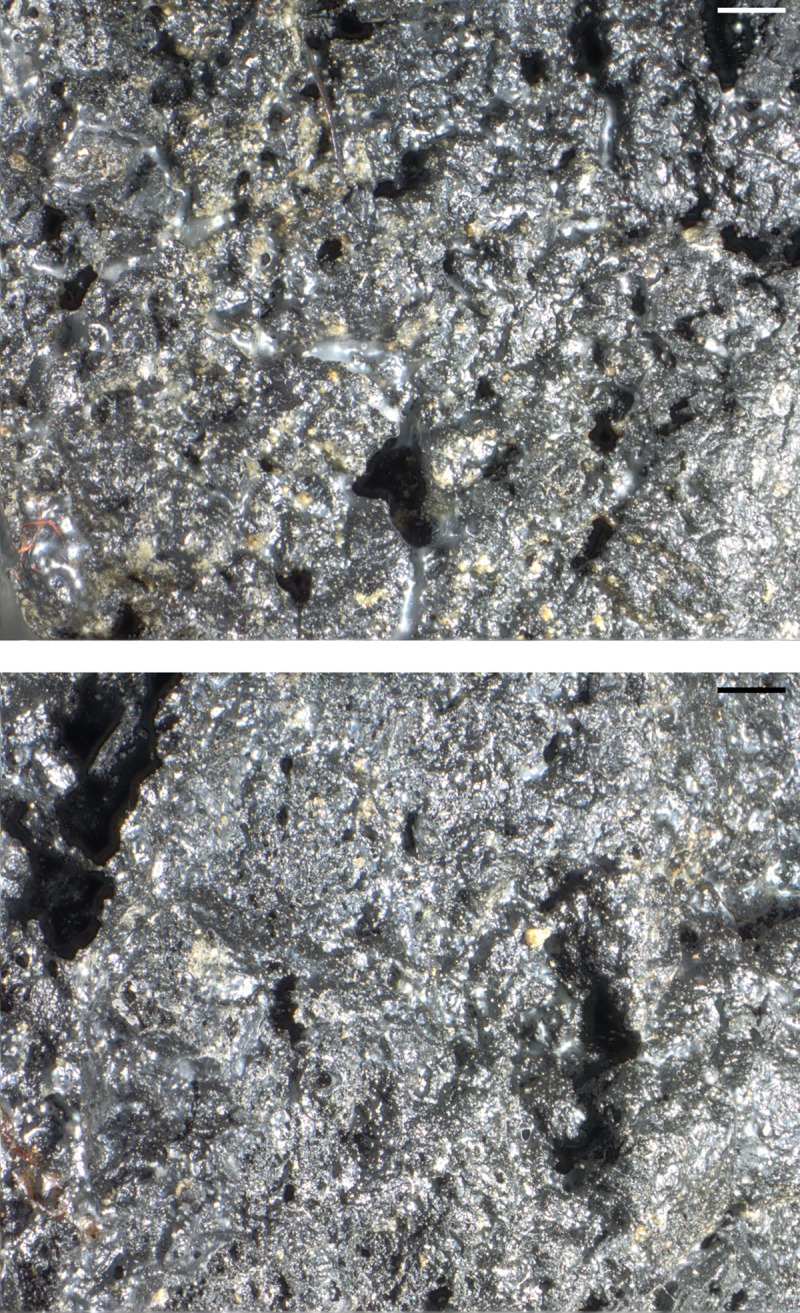
The two fractured faces of sample D under the binocular (10x). Scale bar lengths: 1 mm. Image: ÖAW-ÖAI / A. G. Heiss.

When observed with the naked eye, the rings’ irregularly broken faces appear very dense. Neither pores nor plant parts are visible, but the otherwise compact and seemingly homogeneous matrix is traversed by conspicuous furrows similar to the superficial furrow in sample D. Under low magnification (10x), a few supposed grain fragments as well as the very small pores became visible (Fig 14, Fig 15, Fig 16). None of the three rings showed a differentiation of crumb and crust.

### Plant tissues in sample A

**Fig 17:**
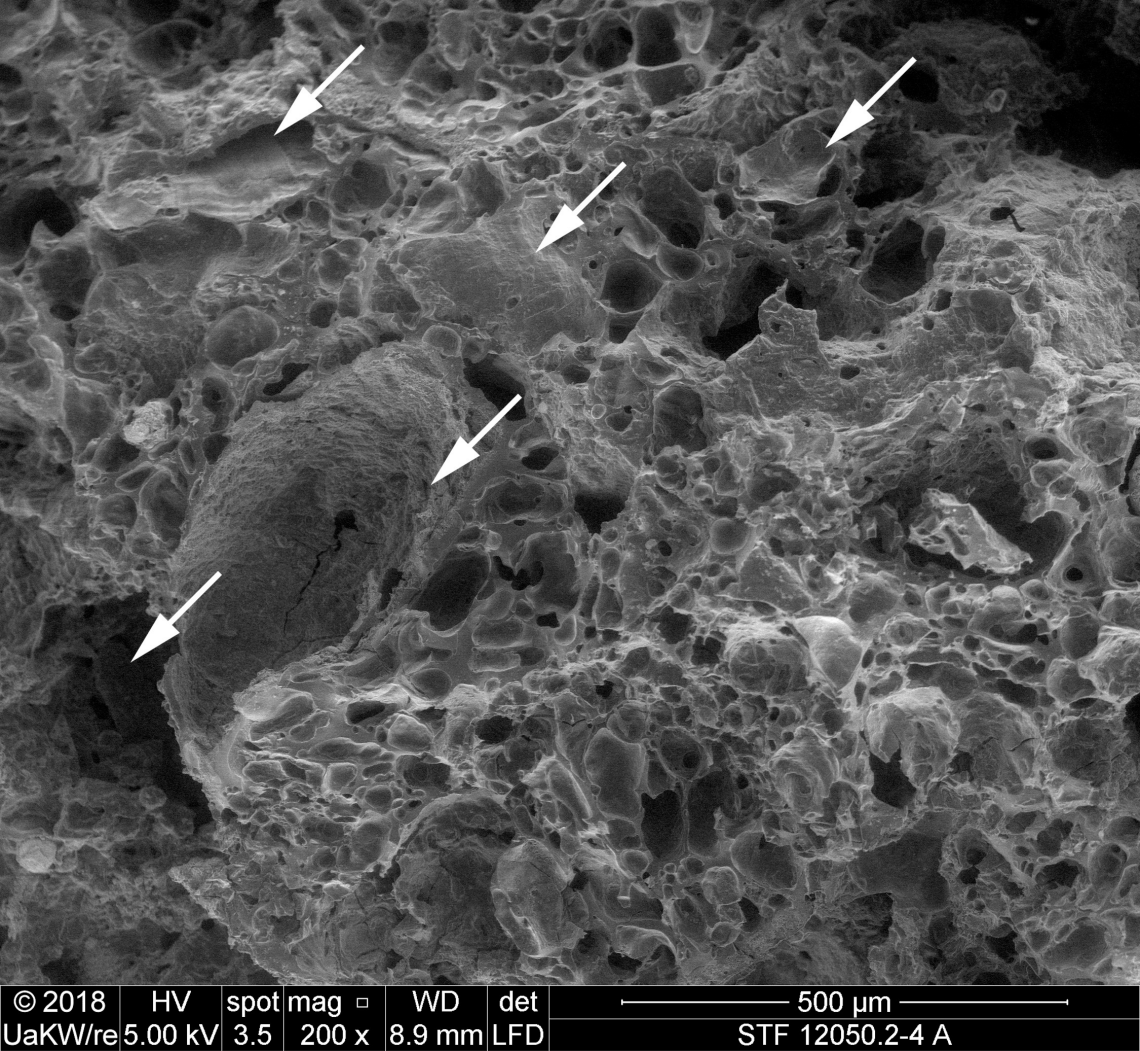
SEM overview of one of the fractured faces in sample A. Arrows indicate cereal bran fragments. Image: die Angewandte / R. Erlach.

**Fig 18:**
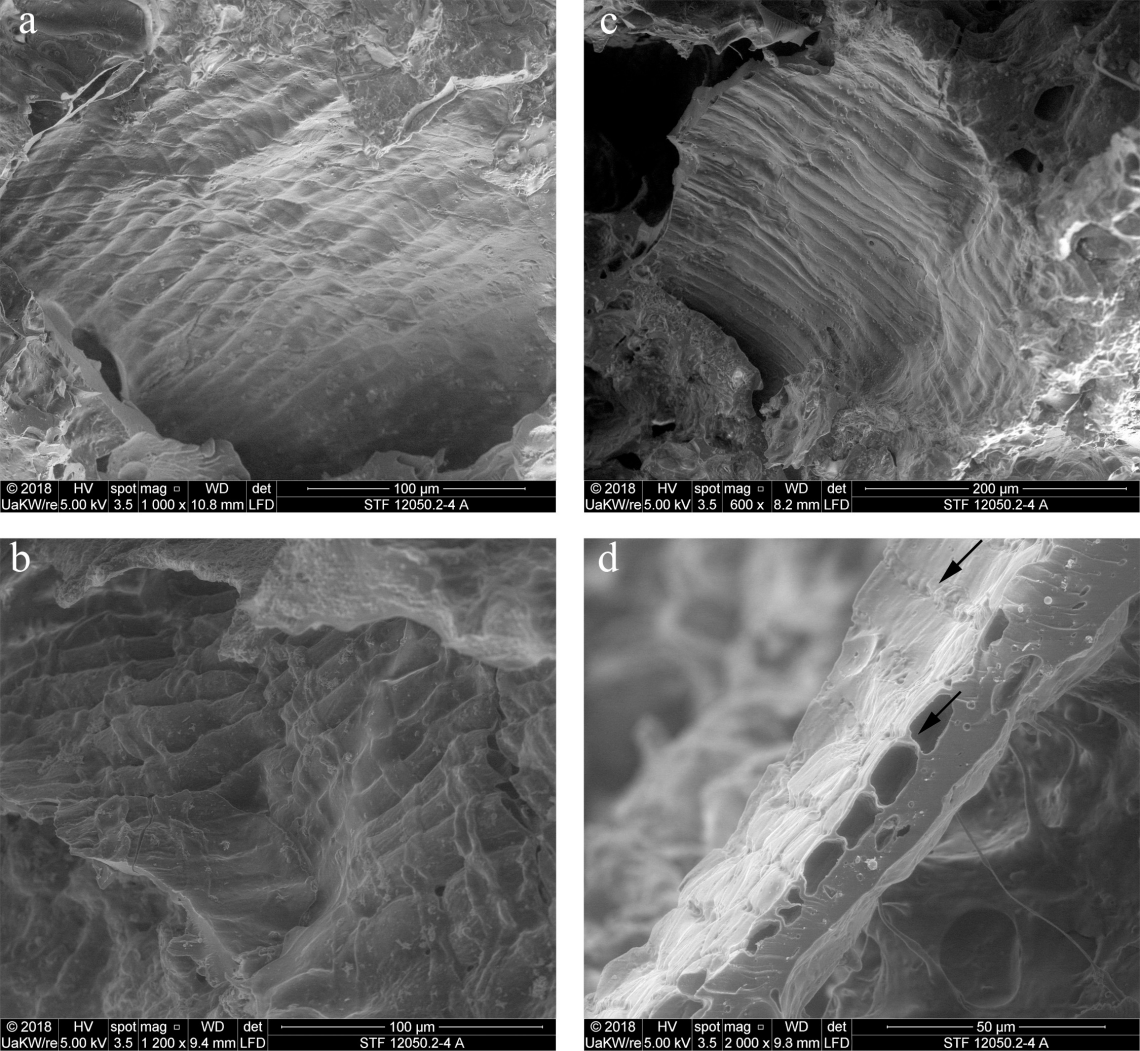
**SEM of patches of transverse cells in sample A**, **a** and **b** with overlaying tubular cells. **a**, **b**, **c**, probably thin-walled transverse cells of the barley type (cf. *Hordeum vulgare*). **d** transverse cells of *Triticum* sp. in cross-section, arrows indicating the conspicuous wall thickenings and “wavy” aspect of the pitted thick walls. Image composed from a stack of 3 images using Helicon Focus 7. Image: die Angewandte / R. Erlach.

**Fig 19:**
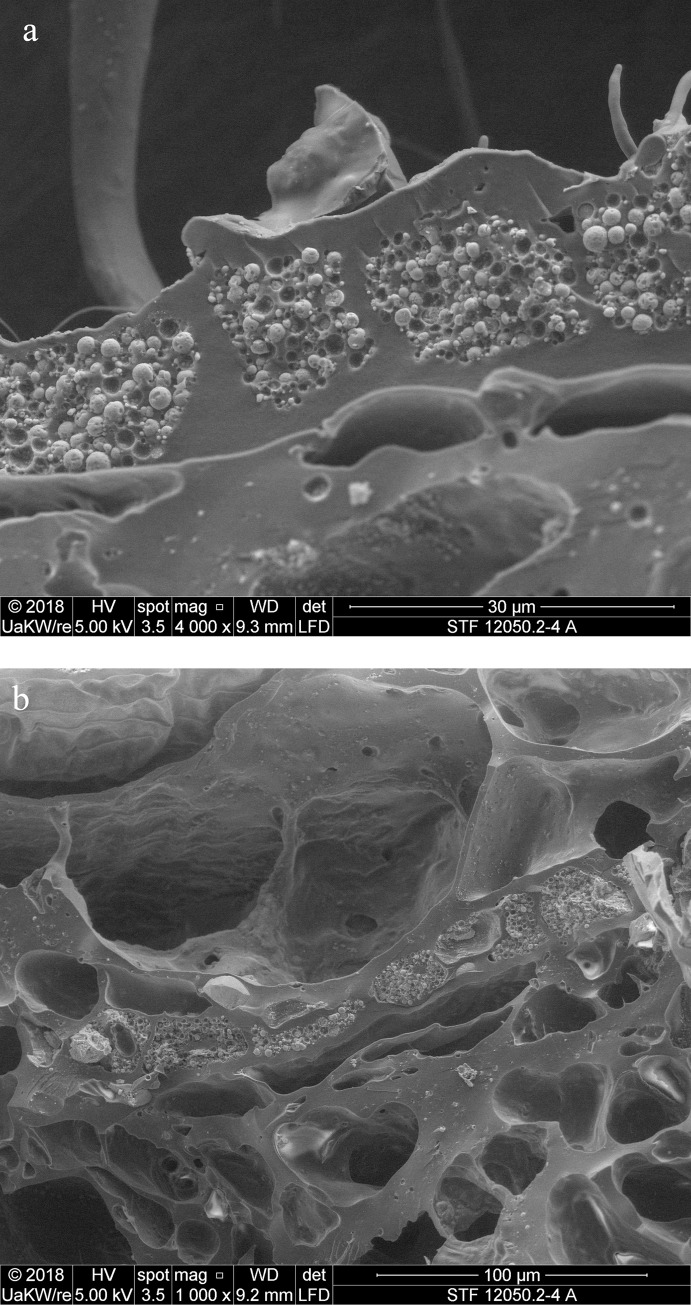
Patches of aleurone tissue (with still intact aleurone granules) in cross-section. The single layer indicates a cereal other than barley. Image: die Angewandte / R. Erlach.

**Fig 20:**
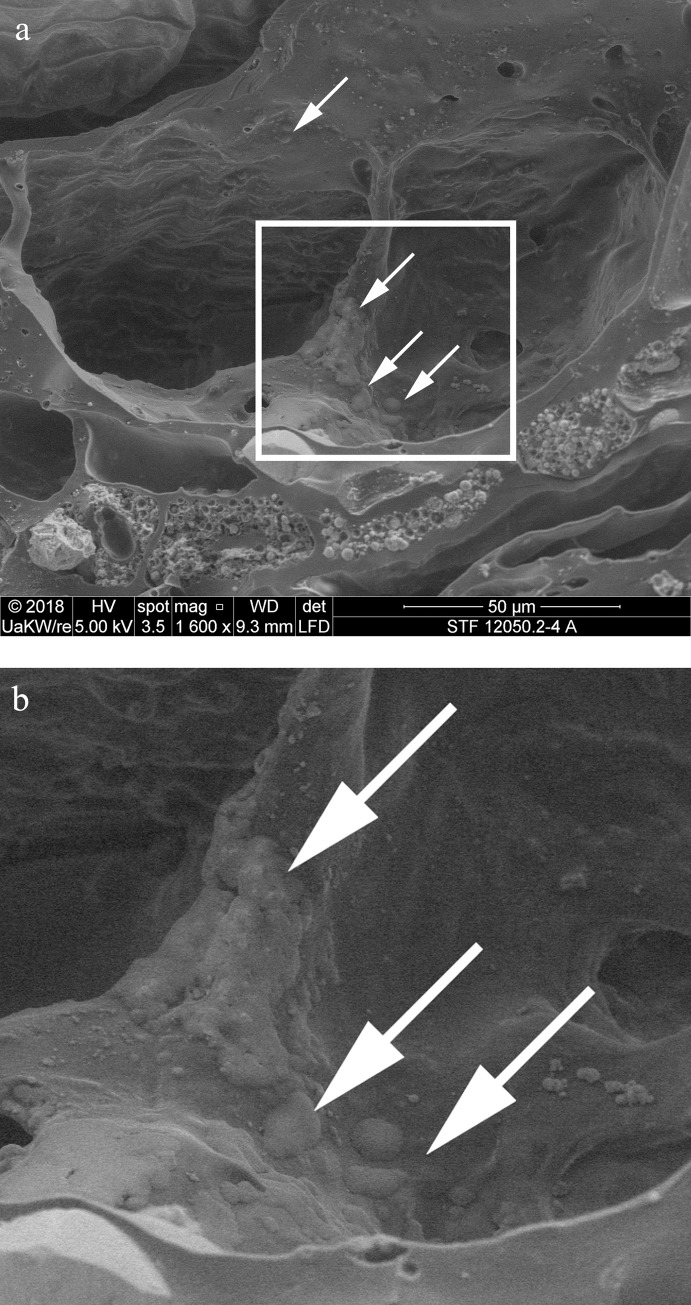
Partly gelatinised starch granules in the endosperm of sample A. **a** Overview and position in relation to the aleurone layer (close-up of **Fig 19B**). Arrows indicate structures resembling incompletely gelatinised starch granules, the rectangle indicates the position of image **b**. **b** Magnified area as indicated in **a**. Image: die Angewandte / R. Erlach.

**Fig 21:**
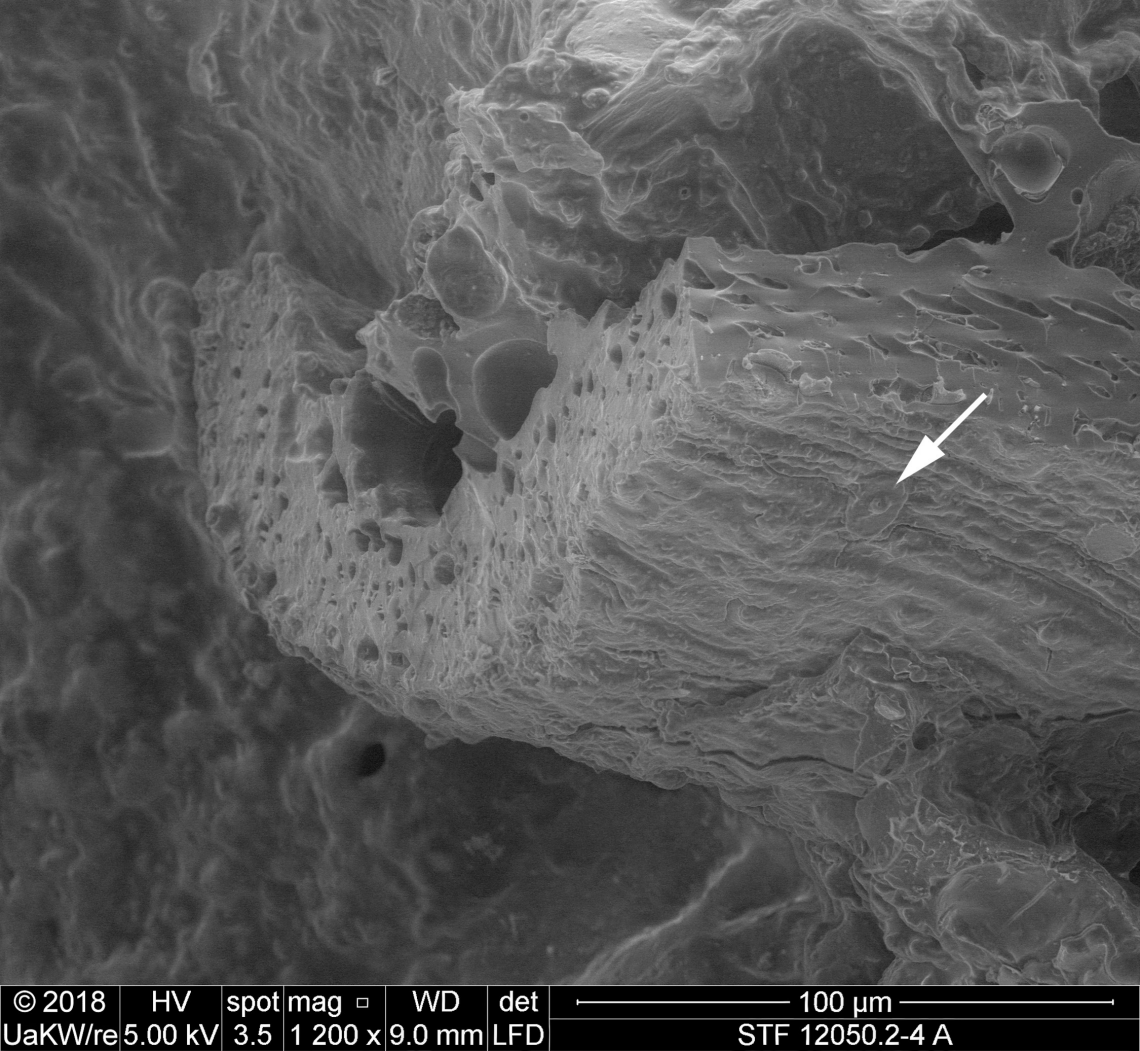
Glume of hulled barley (*Hordeum vulgare*). Image composed from a stack of 3 images using Helicon Focus 7. Image: die Angewandte / R. Erlach.

When observed under magnification (50x – 500x) numerous fragments of cereal bran could be observed (Fig 17) in the porous, starchy matrix of sample A. Here they shall be listed following their arrangement in the cereal grain from the outside inwards: patches of transverse cells, sometimes with tubular cells still adhering (Fig 18), were the predominant bran parts observed. The majority could not be sufficiently identified to genus level in most cases as their cell wall thicknesses could only be observed indirectly. Most of them at least closely resembled barley (cf. *Hordeum vulgare*) transverse cells (Fig 18A–18C). A single fragment was identifiable as *Triticum* sp. (Fig 18D). All observed aleurone tissue was single-layered (Fig 19), therefore giving evidence for some cereal other than barley. Close to former grain surfaces, even a few portions of relatively intact endosperm tissue were found. In some of these endosperm cells, spherical subcellular structures were observed, which we interpret as non-gelatinised or partially gelatinised starch granules (Fig 20). Full gelatinisation of starch granules is the result of (reversible) swelling of starch grains by soaking in water, followed by elevated temperatures of at least 55 to 75°C (see section “Soaking and kneading”). A single fragment of a barley (*Hordeum vulgare*) glume could be identified by the characteristic “double” (crescent-shaped plus orbicular) short cells (Fig 21).

**Fig 22:**
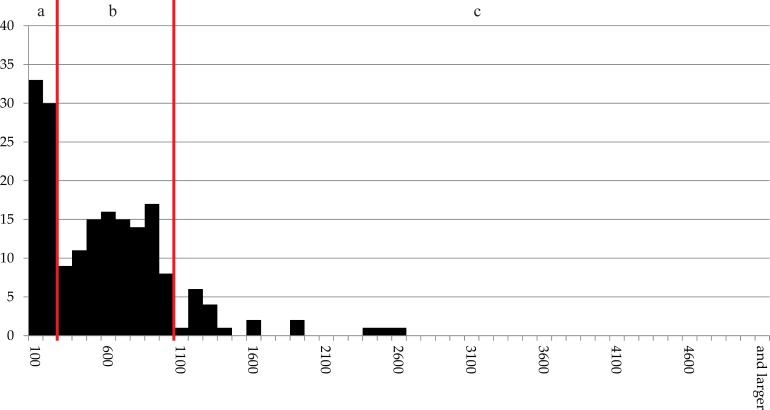
Histogram of the recorded maximum dimensions of components (N = 187) in sample A. X axis: number of occurrences, Y axis: size class in micrometres (100 μm steps). Grouping is according to grain sizes of modern grain products: a) flour and dunst (smaller than 300 μm), b) semolina (300–1000 μm), and c) grist (larger than 1000 μm). Raw data is given in S1 Table.

The distribution of grain sizes of the tissue fragments recorded under light (N = 122) and scanning electron microscope (N = 65) are presented in Fig 22. 86% of the measured particles do not exceed 1 mm.

### Pores

**Fig 23:**
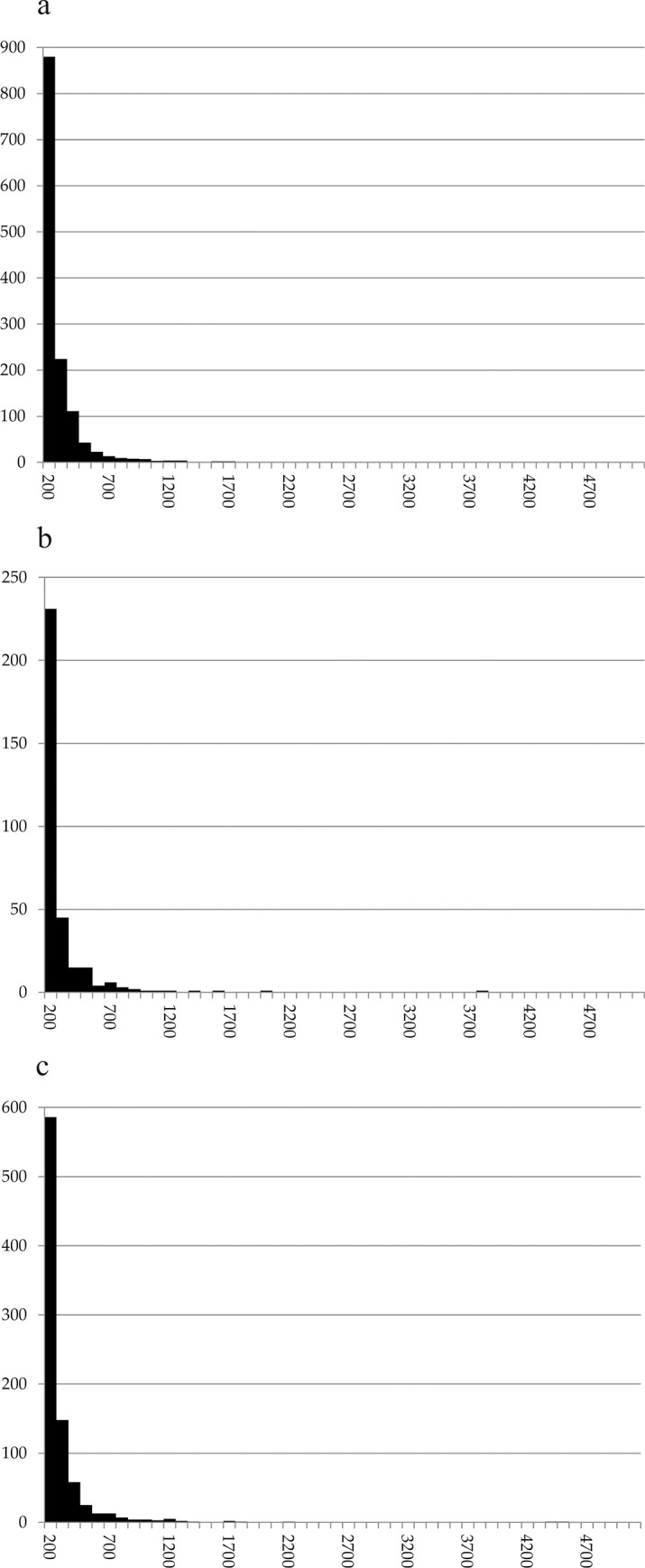
**Histogram of the pores measured in a) sample A (N = 1,341), b) sample C (N = 328) and c) sample D (N = 875)**. X axis: number of occurrences, Y axis: size class in micrometres (100 μm steps). Raw data is given in S2 Table.

All three samples are characterised by very high and nearly identical proportions of micropores, i.e. cavities not exceeding 200 μm in diameter: While in sample A, 66% of the measured pores fall into this category, it is 70% in sample C and 67% in sample D. In all three annular objects, the proportion of pores exceeding 1 mm lies around 2% (Fig 23).

## Discussion

### Ingredients of sample A

Hulled barley is without doubt an ingredient of the bread-like object, as concluded from the evidence of a *Hordeum vulgare* glume remain. Furthermore, a wheat species (*Triticum* sp.) was clearly identified by the characteristic patches of transverse cells. Taking the quite relevant proportions of einkorn (*Triticum monococcum*), spelt (*T*. *spelta*), and Sanduri wheat (“new” glume wheat, *Triticum* cf. *timopheevii*) in the overall cereal spectrum of the settlement [49–51], it is very likely that one of these three glume wheats forms the second constituent of sample A.

The other cereal remains require a bit more caution in their interpretation due to their low discriminatory power: the single-layered non-*Hordeum* aleurone patches may very well derive from the above-mentioned wheat species, but could also be from virtually any other cereal. Likewise, the transverse cells identified as cf. *Hordeum vulgare* leave at least some room for interpretation, as e.g. millet transverse cells look very similar to those of barley [89]. Considering the dominance of *Panicum miliaceum* in the archaeobotanical record of the site, we should therefore at least give a thought to the hypothesis of broomcorn millet being an additional component of the annular object(s). However, millets are difficult to dehusk completely [106], and experience in archaeobotany has shown that at least some fragments of their characteristic glumes [107, 108] are usually preserved in processed cereal preparations [37]. No such evidence was found whatsoever.

As a conclusion, unless future investigations of the rings should result in positive evidence for other cereals, hulled barley and some (probably hulled) wheat species in unknown proportions [10] shall be considered as the cereal ingredients of sample A and quite likely also of the other two charred rings. No evidence for additional plant-based components such as intentionally added condiments [10] or accidentally processed weeds was found. As liquid or soluble components ranging from dairy products to salt cannot be traced via the chosen set of methods, no statements can be made on this topic.

### Involved processes

#### Grinding and sieving (sample A)

Considering the overall grain size distribution as given in Fig 23, 34% of the measured particles would classify as flour or dunst when compared to modern standards [98, 99]. An additional 52% would range within the modern grain size classification as semolina. The remaining 14% remain in the lower range of grist, the largest measured particle hardly exceeding 2.5 mm. If intended for consumption, the flour quality of the three Stillfried rings would be considered as rather fine quality, and comparable to modern wholemeal breads [109]. When comparing the measurements to the (still only scarcely available) extant data from other archaeological finds, sample A corresponds best to find no. 2010.012.2907.5 from the Neolithic site of Parkhaus-Opéra, Zürich, Switzerland [10]. A considerable amount of care [110] has been invested in grinding and sieving the flour used in the production of this ring, and most probably also of the other two annular objects.

#### Soaking and kneading

Mechanical stress during kneading or subsequent drying processes can easily lead to ruptures of doughs with low water contents, which requires extra caution in the kneading of e.g. biscuit [111] or pasta [112] doughs. As in previous archaeological finds [30], the numerous large furrows observed in the charred samples from pit V5400 are interpreted as hints in this direction.

**Fig 24:**
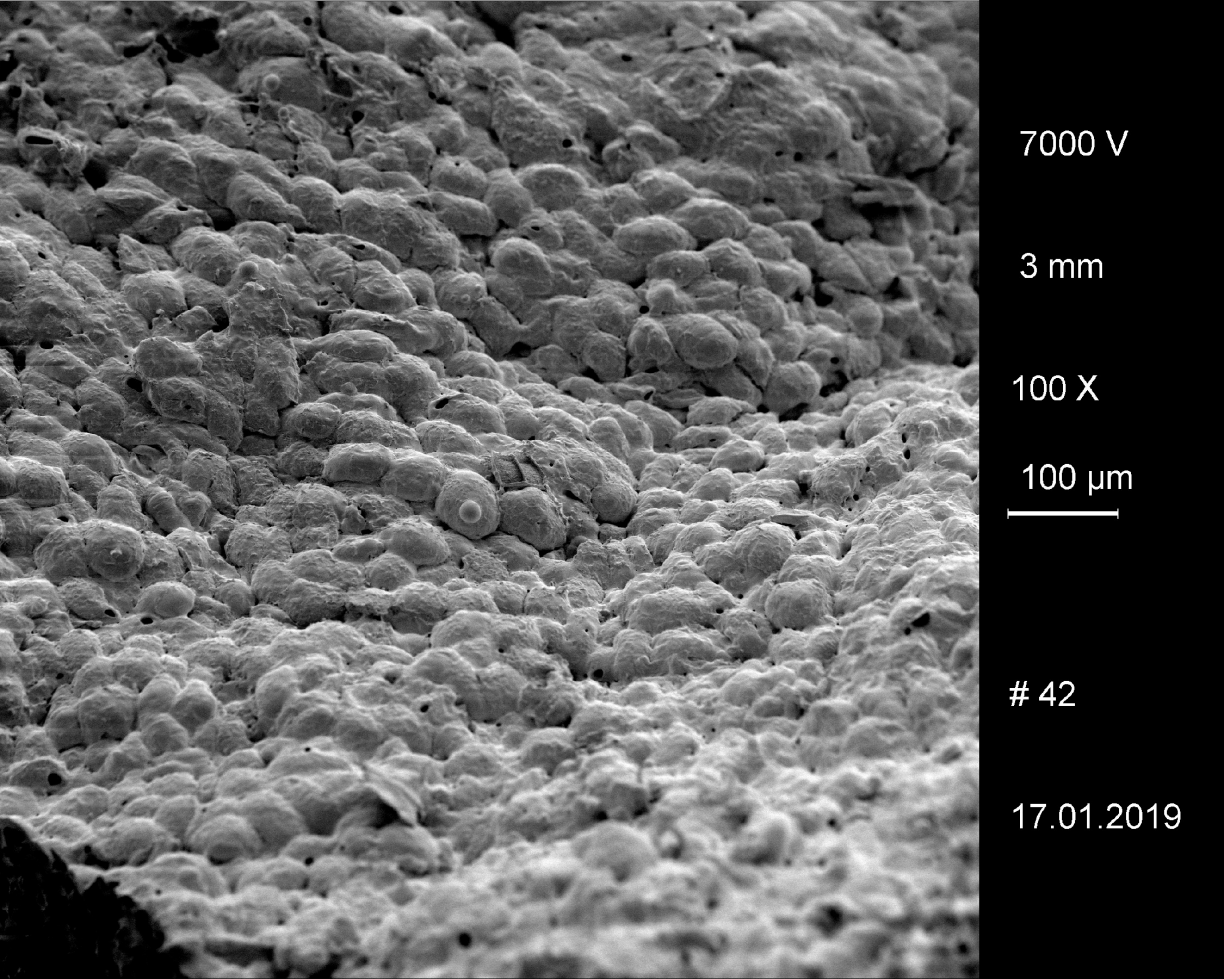
Cross section of charred “*Grünkern*” spelt (immature *Triticum spelta* ‘Bauländer Spelz’). After charring of the dry grains at 230° C for 7 hours, starch granules are still easily discernible. Images: Universität Hohenheim, M. Berihuete Azorín. Images: Universität Hohenheim, M. Berihuete Azorín.

In the SEM micrograph, structures were visible which are interpreted as non-gelatinised or partly gelatinised starch granules. Full gelatinisation of starch granules is the result of (reversible) swelling of starch grains by soaking in water, followed by elevated temperatures of at least 55 to 75°C [113–115]. Experimental studies have demonstrated that the degree of gelatinisation of starch granules is not only preserved in uncharred archaeological remains [115] but also in charred material [116], at least if charred in dry state (Fig 24).

As a conclusion, we assume that the starch granules observed in sample A indicate a cereal preparation either heated at rather low moisture content [117], or not heated at all. The annular objects could therefore have been shaped from uncooked/unbaked dough or grain-paste [118], and were either cooked/baked in dry state, or air-dried. The question still remains whether the partial gelatinisation of starch granules observed in the sample may be an artefact of the charring process or bear some other significance.

#### Fermenting

Modern and historical examples show that there is a wide continuum between the extremes of hard and dense (unfermented) and fluffy (thoroughly fermented) dough. Yet until now, no systematised catalogue of criteria exists to describe the degree of fermentation of archaeological cereal preparations. Key features [119] need to be defined. Extant data from bread research need to be transferred onto charred archaeological finds. These topics are being worked on in within the scope of the project PLANTCULT. For now, and until a database allowing for statistical comparison with experimental data will be available, we have to rely on a comparative approach relating to known finds of archaeological material.

Overall pore size distribution in the Stillfried rings is basically comparable to other cereal products interpreted as non-fermented [10, 30, 120]. In a more detailed comparison with bread-like objects from which pore distribution data have been recorded using the same methodology as in the current paper, the proportion of micropores (66–70%) in the three Stillfried rings is the same as in the sacrificial “cakes” from the Archaic/Hellenistic sanctuary at Monte Papalucio, Oria, Italy (66–82%) [26], but twice as high as in the bread-like object from the Gallo-Roman cemetery of St. Memmie, France (33%) [30] or those from the Neolithic lakeshore settlement Parkhaus-Opéra (26–29%). The proportion of pores exceeding 1 mm (roughly 2% in each of the rings) is comparable to the find of St. Memmie, while measuring the Monte Papalucio finds had not resulted in any values of this size class [26]. Density and degree of fermentation of the Stillfried rings therefore seems to lie somewhere between the aforementioned finds.

It must be stressed that the chosen cut-off value, i.e. omitting pores with diameters under 100 μm, affects the resulting data and its interpretation. We chose the value for two main reasons, the first being that a) any microscope-mounted camera has limited resolution. If counted, single pixels in the image will strongly bias the measurements towards cavities even smaller than micropores [10]. Secondly, b) the authors are convinced that very small pores are consequences of charring and do not contribute to the knowledge of the degree of fermentation of a cereal product.

**Fig 25:**
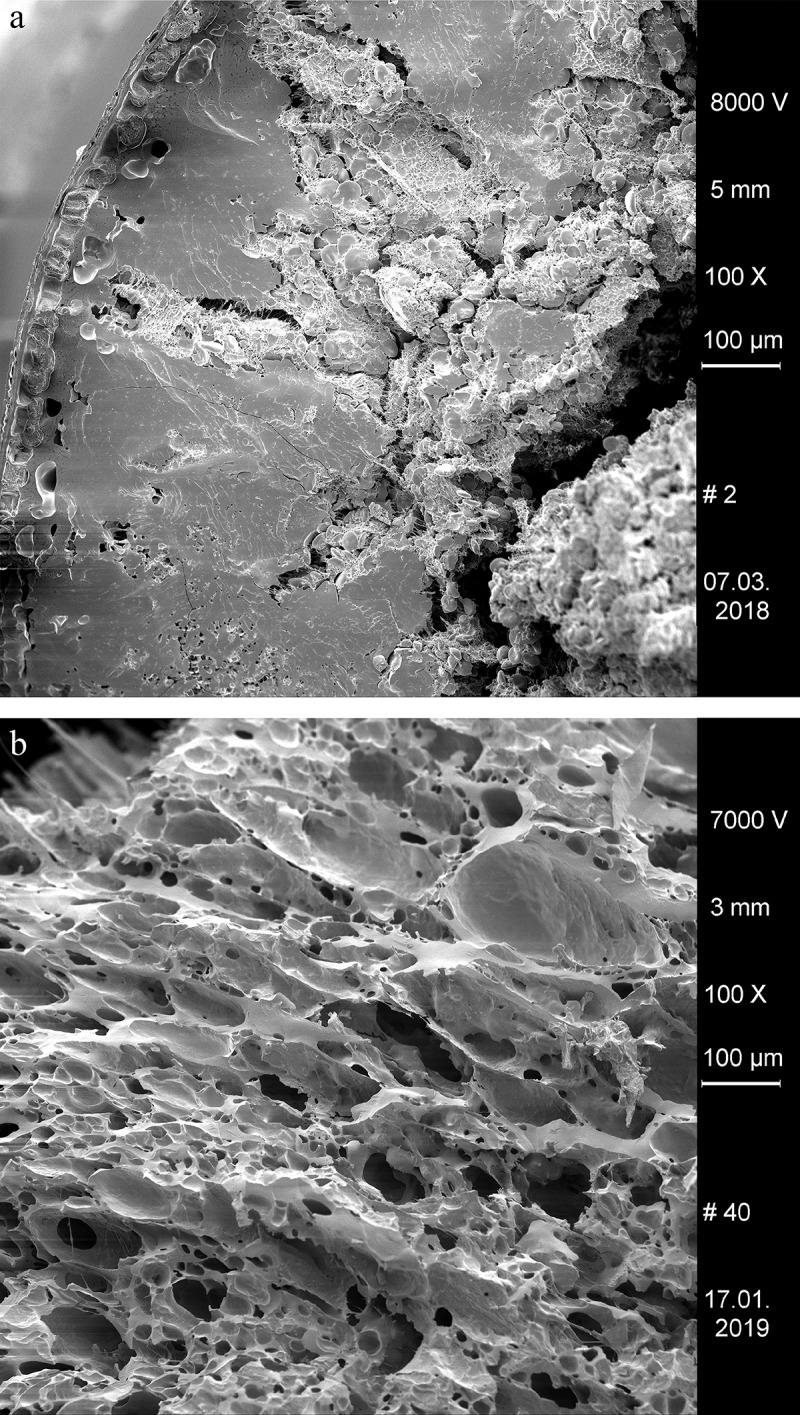
Cross section of charred “*Grünkern*” spelt (immature *Triticum spelta* ‘Bauländer Spelz’). After charring a) at low temperature (230° C) for 7 hours, wide portions of the endosperm have fused into an amorphous glassy matrix while others have formed irregular cavities. Charring b) at high temperature (300° C) for 6 hours has led to extensive formation of cavities with a broad range of sizes, the largest of these “pores” slightly surpassing the category of micropores. Images: Universität Hohenheim, M. Berihuete Azorín.

The reason for this shall briefly be highlighted: charring leads to the formation of pyrolysis gas predominantly of carbon dioxide, carbon monoxide and methane [121, 122], as well as water vapour. The latter originates from both residual bound water and from the chemical breakdown of starch [123]. Together, the expanding gases inevitably lead to the formation of cavities in the intermediate liquid phase of biopolymers breaking down [124]. The irregular bubbly structures found in most archaeological finds of intact cereal grains may be taken as examples for this process of cavity formation. This also occurs in grains experimentally charred at elevated temperatures (Fig 25).

It must be stressed that even higher cut-off values than the ones applied in the current and previous studies may be required in the future. Experimental work within the scope of the project PLANTCULT is ongoing, but preliminary results already suggest that great caution is required in the interpretation of pore sizes, in particular when using smaller pores, or even micropores, as a diagnostic character for the presence of (leavened) “bread”.

#### Shaping

The aforementioned furrows indicate an overall low water content in the dough during preparation. The deep length-wise furrows extending to the surfaces of all three annular objects may directly derive from the process of rolling the dough into a tube before connecting its ends into a ring-like shape. It is, however, important to point out that no remains of such connective points are preserved.

#### A ring is a ring is a ring. Daring a description

As with fermentation in particular, the general criteria for the classification of archaeological finds of cereal preparations are still in an early stage. Extant pioneering work requires 1) refinement, 2) an increase in complexity of characters, and 3) general applicability. Taking the criteria proposed for the Çatalhöyük material as a reference, for example, the rings from Stillfried show traits comparable to the published Matrix Types 2 and 4 [102], resembling a finely ground porridge. Combining the features of 1) a rather unfermented dough and 2) the raw state before charring with 3) the low initial water content, we would consequently categorize the material of the Stillfried rings as air-dried dough [118] made from finely ground barley and wheat flour.

Using modern cereal preparations as references, the rings’ *chaîne opératoire* is most similar to that of modern pasta, or of *tarhana*/*tarhonya*/*trahanas* [125–127], while they roughly share shape and size with Italian *tarallini* [26] and Russian *sushki* [128]. Unless a better solution for a terminology is found, we propose to limit denominations of archaeological cereal preparations to this approach, i.e. to give separate comparative descriptions for components, shape, and making.

### Further interpretative approaches

#### Taphonomy of the find assemblage

In her review article, T. Popova states “It has been established that bread fragments are among the most common finds in ceremonial/ritual fireplaces, burial mounds, and sanctuaries (…)” [129]. This is statement is certainly correct, but in the authors’ opinion it should by no means be understood by the reader as a postulate of a scarcity of “bread” use in “profane” contexts. Cereal products are indeed a very frequent archaeological find category, yet due to their fragility they are highly prone to fragmentation [130, 131], with their fragments usually not allowing for any inference to the original artefact such as bread in the broadest sense, porridges, and the like [118, 125, 132]. Even worse, as D. Fuller stated, they are also “more often than not set aside by archaeobotanists as indeterminate fragments” [131].

The reason for the seemingly rare occurrence of intact “bread” in such ritual contexts may simply lie in taphonomical processes, and of sample treatment; aside from ritual depositions [26, 30, 133, 134], large numbers of more or less intact charred bread-like objects also originate from lakeshore settlements [10, 135–137] and from burned-down bakeries [138, 139]. These find situations share the common trait of reduced mechanical stress affecting the fragile charred finds, either because the objects a) had been deliberately and carefully deposited, b) were embedded in soft lakeshore sediments, or c) were charred and embedded *in situ*. Furthermore, all these finds have in common that they are usually grab finds: the destructive potential which flotation can have on charred plant material [140] has just recently been reconfirmed [141].

Were we to interpret the genesis of the pit’s secondary filling against the background of an entirely “profane” scenario versus one also involving ritualised (“ceremonial/cultic”) activities, one of the key differences in their respective taphonomies would concern the way the finds were deposited: disposing of the debris of a burnt-down house and using it as the backfill for a no longer used storage pit would probably not involve much care, in contrast to a structured and ritualised process [142, 143]. Taking into account these considerations together with the “odd” inventory of the pit, as well as the rather good state of preservation of the three annular bread-like objects from pit V5400, may indicate careful deposition and serve as a indication of a ritual/cultic interpretation of the filling process.

If we accept deliberate deposition of the objects in pit V5400, this inevitably begs the question of the purpose of the dough rings themselves: do they represent “everyday food” deposited in the pit? Were they made for a certain symbolic purpose?

#### Modern dough rings

In general, historical and ethnographic sources document a rich wealth of ring-shaped breads and pastry across Europe. The most prominent and best known ones today are indeed “everyday food”, sharing the characteristic of leavened dough which is boiled before baking, such as the *bagel* of the Polish Jews, documented since early Modern Times [144] and its Russian counterparts such as the *sushki* [128]. The *simit* in Turkey and the *koulouria* in Greece, both supposedly of Ottoman origin [145], are produced the same way.

In contrast, other types of ring-shaped cereal products are documented as distinct elements of specific festive and ritual contexts, such as the early Christian *corona* [33] of unknown components and production traits. The *sa pertusitta* is a traditional baked ring in Sardinia’s New Year’s Eve [146], just as southern Italy’s *raffiuoli*–a particular sweet variant of *taralli*–are produced for religious feast days [147, 148]. For the days around Christmas and New Year, the Alpine region knows deep-fried annular *kiachl* [149], while Styrian [150] and Lower Austrian [151] folklore includes *Beugel*, a variant of the bagel, during carnival. Greek *kerkelia* are produced for Lazarus Saturday by Pontic Greeks [152]. The kolač, in its innumerable spelling variants and preparation techniques, is part of religious feast days throughout Slavic countries [153, 154] and beyond. It should be mentioned, however, that the Romanian [155] and Moldvian [156] *colaci* have their principal purpose in serving as offerings for the dead [157].

All of the aforementioned examples only share a roughly annular shape as common character, yet they are produced by following strongly diverging *chaînes opératoires* and intended to serve a wide range of different purposes. Consequently, the ring shape alone is an insufficient interpretive element for elucidating the nature of the Stillfried rings.

#### Archaeological finds

If we consider archaeological finds of ring-shaped “bread” in the widest sense across Europe, i.e. bread-like objects, we find that the evidence is generally very limited. Find no. 2010.012.2285.3 from the late Neolithic site of Parkhaus-Opéra might be the oldest documented find from central Europe to date, but it is of unknown purpose and unspecific find context [10]. Several ring-shaped “biscuits” are documented as offerings in an Archaic/Hellenistic sanctuary in southern Italy [134] which have recently been revised [26]. Finally, there is thorough analysis and documentation available for a large number of annular bread-like objects, some on metal strings, as grave goods in Viking Age cremation graves in Sweden [133].

The annular object from a Late Iron Age cremation grave at Wederath in Germany [158], initially published as “ring cookie” (*Ringgebäck*) [159, 160], has unfortunately been proven recently to be entirely made of beeswax [161].

#### Possible significance of the Stillfried rings

In comparison to the other cereal preparations (Table 1) from Stillfried an der March which have already been published [49], the three annular objects from pit V5400 differ in both their shape and the production processes involved. Their finely ground flour and intentional shaping certainly indicate that more time was invested in their production than necessary for subsistence. The rings may therefore have been of higher value than the other cereal preparations described from Stillfried.

The overall “odd” assemblage [45] in the secondary filling of pit V5400 may or may not be a valid argument against the dough rings representing everyday food. In the authors’ opinion, the strongest point lies in the context of clay rings and dough rings deposited together which seems highly unlikely to have happened by sheer chance. Like similar finds from synchronous sites in the region [162], the clay rings are interpreted as loom weights [47, 163], and they bear actual traces of use.

Any practical use for the dough rings found in context with them is hard to imagine. We therefore suggest the hypothesis that the dough rings were created on purpose as imitations of the clay loom weights of the same shape and probably were not produced for consumption in the first place. The exact purpose of the Stillfried cereal rings remains unknown, as well as the time of their charring–during the catastrophic fire which destroyed the house, or within the course of the ritualised filling of the pit.

No literature on directly comparable finds was available to the authors, yet some approximations shall be attempted; to a certain extent, cultic aspects of loom weights have frequently been postulated and discussed, and there are numerous examples of intentionally deposited loom weights–annular, truncated pyramid shaped, or irregular–in central European Late Bronze Age settlements [162], and likewise as grave goods. The latter are, however, usually miniatures, and they all resemble the truncated pyramid type. Archaeological finds of dough imitations of loom weights are, however, entirely unknown up to now–in the view of all the adverse preservation conditions for processed cereals [10] and even more so for entire bread-like objects (see above), this is not entirely surprising.

## Conclusions

The three annular charred finds from former storage pit V5400 at Late Bronze Age Stillfried were made of dough from rather finely ground wheat and barley meal which was most likely air-dried or baked at low temperature prior to charring.

Comparison to the extant data on food preparations at the site, combined with the “odd” find context strongly suggest that the three annular objects had most likely not been produced for consumption, but had rather served some specific purpose prior to or during the deposition of the remainders of the burnt house in the pit.

In view of the general difficulties in differentiating “profane” domestic refuse from remainders of ritual activities at all–or rather, of highlighting both aspects if present at the same time, the role of food preparations in these past activities is even more difficult to investigate. Without favourable preservation conditions, thorough sampling and in-depth scientific investigations, such organic remains may simply go unnoticed in the archaeological record.

In order to recognise and preserve such “odd” objects, we strongly recommend direct sampling of charred plant concentrations, especially in “odd” contexts. Avoiding not only exposure to mechanical tensions but also to flotation seems to be crucial for gaining all available information on cereal food preparations.

## Supporting information

S1 Table Particle measurements of sample A. Raw data of the histogram in Fig 21.
(XLSX)

S2 Table Pore measurements of the three annular objects. Raw data of the histograms in Fig 21.
(XLSX)
